# New insights into the structures and interactions of bacterial Y-family DNA polymerases

**DOI:** 10.1093/nar/gkz198

**Published:** 2019-03-27

**Authors:** Kęstutis Timinskas, Česlovas Venclovas

**Affiliations:** Institute of Biotechnology, Life Sciences Center, Vilnius University, Saulėtekio 7, Vilnius LT-10257, Lithuania

## Abstract

Bacterial Y-family DNA polymerases are usually classified into DinB (Pol IV), UmuC (the catalytic subunit of Pol V) and ImuB, a catalytically dead essential component of the ImuA-ImuB-DnaE2 mutasome. However, the true diversity of Y-family polymerases is unknown. Furthermore, for most of them the structures are unavailable and interactions are poorly characterized. To gain a better understanding of bacterial Y-family DNA polymerases, we performed a detailed computational study. It revealed substantial diversity, far exceeding traditional classification. We found that a large number of subfamilies feature a C-terminal extension next to the common Y-family region. Unexpectedly, in most C-terminal extensions we identified a region homologous to the N-terminal oligomerization motif of RecA. This finding implies a universal mode of interaction between Y-family members and RecA (or ImuA), in the case of Pol V strongly supported by experimental data. In gram-positive bacteria, we identified a putative Pol V counterpart composed of a Y-family polymerase, a YolD homolog and RecA. We also found ImuA-ImuB-DnaE2 variants lacking ImuA, but retaining active or inactive Y-family polymerase, a standalone ImuB C-terminal domain and/or DnaE2. In summary, our analyses revealed that, despite considerable diversity, bacterial Y-family polymerases share previously unanticipated similarities in their structural domains/motifs and interactions.

## INTRODUCTION

Bacterial DNA replication machinery has evolved to copy undamaged DNA in a fast and accurate manner (1). However, DNA lesions originating from genotoxic environmental sources such as UV light or chemicals may cause obstacles for replicative polymerases. Eventually, this leads to the formation of a single-stranded (ss) DNA gap that acts as a distress signal to the cell. The signal induces extensive transcriptional changes known as the SOS response which, although complex, depends on only two proteins, RecA and LexA (2). The RecA recombinase polymerizes on the ssDNA regions to form a RecA-ssDNA filament, in turn stimulating the autoproteolytic cleavage of the transcriptional repressor LexA. The self-cleavage of LexA leads to de-repression of multiple genes including those involved in DNA repair. In the early SOS response, the induced proteins promote accurate DNA repair through homologous recombination. However, if DNA damage persists, bacteria turn to mutagenic DNA repair as a last-ditch effort to complete DNA replication. DNA is replicated across the lesions during a process called translesion DNA synthesis (TLS). TLS is performed by specialized DNA polymerases, most of which belong to low-fidelity Y-family DNA polymerases (3,4). Like other right-handed polymerases, Y-family members have palm, fingers and thumb domains. They also have an additional domain, usually referred to as the little finger (LF) domain in bacteria or the polymerase-associated domain (PAD) in eukaryotes. Y-family DNA polymerases have more open active sites and thus can more easily accommodate bulky DNA lesions (4). Furthermore, they lack the 3′→5′ exonucleolytic proofreading activity typical of high-fidelity DNA polymerases.

Bacterial Y-family DNA polymerases are generally divided into two branches typified by *Escherichia coli* DinB (Pol IV) and UmuC, the catalytic subunit of Pol V (3). *Escherichia coli* Pol IV and Pol V are both induced as part of the SOS response, but their compositions and functional properties differ. Whereas Pol IV corresponds to a single DinB subunit, Pol V corresponds to the UmuD'_2_C complex, which is assembled in multiple stages (5,6). Newly synthesized UmuC is transiently sequestered at the bacterial inner cell membrane. UmuD is synthesized as a homodimer which, in the presence of the RecA nucleoprotein filament, undergoes autocatalytic cleavage to generate UmuD'_2_. The latter interacts with UmuC to form the heterotrimeric Pol V (UmuD'_2_C) that is released into the cytosol. However, Pol V has little TLS activity on its own. Only upon transfer of a RecA subunit along with ATP from the 3′-proximal end of the activated RecA nucleoprotein filament to Pol V, the fully active Pol V mutasome (Pol V Mut) is formed (7,8). Pol V Mut has an intrinsic DNA-dependent ATPase activity that is unrelated to the ATPase activity of RecA nucleoprotein filament (9). ATP is required for Pol V Mut to bind primer-template DNA, whereas the ATP hydrolysis triggers dissociation. In other words, the intrinsic ATPase activity limits the time spent by the Pol V Mut complex on the DNA. Apparently, Pol V is regulated so tightly because it is highly mutagenic and responsible for UV-dependent and most chemical-induced mutations (10). If unchecked, the mutagenic activity of Pol V would be deleterious to the cell. Decades of intense research have revealed the complex levels of Pol V regulation in increasing detail. However, the lack of experimental 3D structures of Pol V or even its catalytic subunit, UmuC, preclude the comprehensive understanding of the interactions relevant to the assembly and mechanism of Pol V. UmuC and DinB share a conserved Y-family core, therefore, the core structure of UmuC could be derived by homology modeling (11,12). However, unlike DinB, UmuC has an additional C-terminal extension that has no obvious relationship with known structures. This C-terminal extension is clearly important for the assembly of active Pol V as it has been implicated in binding both UmuD' and RecA (13–15). Interestingly, DinB can also form a complex with RecA and UmuD (16). However, this complex is markedly different from Pol V. First, since DinB does not have a C-terminal extension, the interactions are mediated only by the polymerase region. Second, the complex formation reduces the mutagenic activity of DinB (16).

The division of Y-family DNA polymerases into DinB and UmuC branches is not always straightforward, indicating that there might be significant differences from the *E. coli* model. This can be exemplified with *Bacillus subtilis*, the model gram-positive bacterium, which possesses four Y-family DNA polymerases. Two of these, YqjH (PolY1) and YqjW (PolY2), are encoded chromosomally, while the other two, UvrX and YozK-YobH, are part of integrated prophages (17–20). Initial phylogenetic analysis placed YqjH into the UmuC branch (3), whereas a subsequent study assigned YqjH as a DinB ortholog (17). On the other hand, YqjW, UvrX and YozK-YobH have been consistently classified as gram-positive UmuC-like proteins and, similarly to *E. coli* Pol V, all three were predicted to be under the LexA control (3,17). However, no UmuD homologs have been found in *B. subtilis*. Therefore, it was suggested that y*qjX*, y*olD* and y*ozL*—each forming a putative operon with one of the three *umuC*-like genes—might represent functional *umuD* analogs (17). Experimental studies revealed that *yqjW* and *yqjX* are indeed induced upon SOS activation, reinforcing the parallels with *umuC* and *umuD* (21). However, attempts to establish whether *yqjX* is required for *yqjW* function produced inconclusive results (19). Studies of DNA damage survival and UV-induced mutagenesis, the hallmark of Pol V function, reported the contribution of both chromosomally encoded *B. subtilis* Y-family polymerases, YqjW and YqjH, albeit with some conflicting results (19,20,22,23). Also, both polymerases were found to play important roles in protecting sporulating cells from DNA damage and in increasing UV-induced mutagenesis (24,25). Thus, *B. subtilis* YqjH and YqjW do not appear to be strict functional homologs of *E. coli* DinB and UmuC (26). Whether YqjW functions *in vivo* as part of a Pol V-like multisubunit complex or as a single polypeptide chain also remains an open question.

DinB (Pol IV) proteins in bacteria are ubiquitous, whereas the prototypic *E. coli umuDC* system (Pol V) responsible for SOS-induced mutagenesis is not. Instead of Pol V, many different bacteria carry a LexA-regulated gene cassette *imuA-imuB-dnaE2*, encoding correspondingly a RecA homolog (ImuA), a catalytically inactive Y-family DNA polymerase (ImuB), and a second copy of the Pol III α-subunit, DnaE2 (27,28). Early studies in *Mycobacterium tuberculosis* and *Caulobacter crescentus* implicated *dnaE2* in induced mutagenesis (29,30). The roles of individual components were clarified in a comprehensive study performed in *M. tuberculosis* and *M. smegmatis* that encode a split *imuA'-imuB/dnaE2* cassette (31). The study confirmed that DnaE2 (also referred to as ImuC (28)) is a mutagenic DNA polymerase requiring the presence of both ImuA' and ImuB for its function (since ImuA' and ImuA belong to the same family, throughout the paper we will use ‘ImuA’ to denote both). ImuB has a C-terminal extension, which binds both DnaE2 and ImuA, and is also essential for the functioning of the mutasome (31). Interestingly, a computational study has found that homologs of the ImuB C-terminal region may also occur as standalone proteins associated with various SOS-response networks (32). However, the 3D structure of either full-length ImuB or even its C-terminal region remains unknown, hampering the understanding of how ImuB interacts with its partners and how these interactions enable mutasome function.

To better understand the structures and interactions that govern the activities of bacterial Y-family DNA polymerases, we performed a detailed computational analysis. We found that the UmuC C-terminal extension has a short region homologous with the RecA N-terminal fragment, itself involved in RecA filament formation. Based on this observation, we proposed a structural model for the UmuC–RecA interaction that rationalizes prior experimental observations. Moreover, we identified similar regions of homology to RecA in a large number of diverse Y-family members, suggesting a prominent role for RecA in regulating bacterial Y-family polymerases. We further explored the possible composition of Pol V analogs in gram-positive bacteria and identified compelling similarities with *E. coli* Pol V. We also identified putative variants of the ImuA-ImuB-DnaE2 system as well as additional domains and sequence motifs apparently involved in protein–protein interactions. Based on our results, we propose that different bacterial multisubunit complexes involving Y-family members have much more in common than previously appreciated.

## MATERIALS AND METHODS

### Bacterial genomes, protein sequence data and homology searches

Non-redundant annotated complete bacterial genomes and corresponding proteomes were obtained from NCBI (ftp://ftp.ncbi.nlm.nih.gov/genomes/). Only genomes labeled as ‘representative’ or ‘reference’ (1568 in total) were included in the representative set. Accession numbers and other relevant data for the complete genome dataset are provided in Supplementary Table S1.

Y- and C-family DNA polymerases, YolD, UmuD, ImuA and ImuB-C protein homologs, were identified in a cumulative database of bacterial proteomes using PSI-BLAST (33). This was achieved in two steps: well-known representatives of each analyzed protein family were used as queries in the first step; all identified homologs were then used as queries in the second step. PSI-BLAST searches were run for up to five iterations with an E-value cutoff of 1e-3. Prior to any searches, the query protein sequences were trimmed to include only representative domains (e.g. only the polymerase domain for DNA polymerase queries). The obtained sets of homologs were then cleaned up by discarding sequence fragments and false-positive matches. The latter were determined by first identifying outliers with CLANS (34) and then verifying their homology to the query protein using HHpred server (35). Prior to any analysis, all intein sequences, identified from multiple sequence alignments and verified using HHpred, were excised. C-family polymerase sequences that were split through an intein were concatenated after its removal.

Positions of genes of all identified proteins in their respective genomes were recorded and were then used to detect any putative operons (e.g. *umuD-umuC*).

### Multiple sequence alignment

Multiple sequence alignments were generated with MAFFT (36) using the accuracy-oriented mode, L-INS-i. Alignments were analyzed, visualized and edited using Jalview (37).

### Sequence classification and phylogenetic analysis

Full length Y-family polymerases were clustered and classified according to their global sequence similarities using CLANS. Sequence groups were discerned by gradually decreasing cutoff *P*-value and assigning groups to split-off clusters. A small fraction of sequences was not assigned to any group.

Phylogenetic trees were constructed with IQ-TREE (38), using Le and Gascuel (LG) substitution model (39). A total of 1000 replicates of ultrafast bootstrap (40) and SH-like approximate ratio test (41) were calculated for evaluating branch support. The initial multiple sequence alignments were truncated to contain only domains common for all sequences. Poorly aligned sequences were discarded (based on visual inspection of the alignment) to improve resulting tree quality. Alignments were then trimmed to contain only positions with fewer than 10% gaps using trimAl (42) to further increase signal-to-noise ratio. The trees were visualized and annotated using iTOL (43).

### Analysis of sequence features

Genesilico metaserver (44) was used for initial analysis of representative protein sequence properties, such as secondary structure and disorder. Secondary structures were predicted with PSIPRED (45) using Uniref50 protein database (46) for profile generation.

Conserved structural domains/motifs were identified based on their alignment to known representative domains/motifs in multiple sequence alignments. Uncertain cases were verified by searching Pfam (47) and PDB databases using HHpred server.

Sequence motifs were visualized using WebLogo 3 server (48). Each sequence motif was visually located (based on motif identification results) and cut out from multiple sequence alignment generated for the analyzed sequence group. Gapped positions were removed based on selected representative sequence for the motif.

To identify any additional domains in C-terminal parts of Y-family members, only sequence regions following the β-clamp binding motif or corresponding sequence position (if no identifiable motif could be detected) were retained. These sequence regions were then used as queries for HMM-HMM searches against PDB and Pfam databases using HHsearch (49) from HHsuite package. The HMM profiles for the sequences were generated by performing two search iterations through uniclust30 database (50) using HHblits (51) from HHsuite package. All three databases were downloaded in June 2018. For selected representative sequences, user-generated multiple alignments were used as queries for the HHpred server.

### Protein structure modeling and analysis

HHpred server was used to identify best structural templates and to generate sequence–structure alignments for modeling. 3D models were generated with Modeller (52). If model quality was deemed insufficient, I-TASSER (53) and ROBETTA (54) servers were used to generate additional models. Model quality evaluation was performed with VoroMQA (55). The models were also inspected visually for any flaws. Structure visualization and analysis were performed using UCSF Chimera (56). Protein 3D structure comparison was performed using Dali server (57).

## RESULTS

### Bacterial Y-family DNA polymerases comprise multiple subfamilies

**Figure 1.**
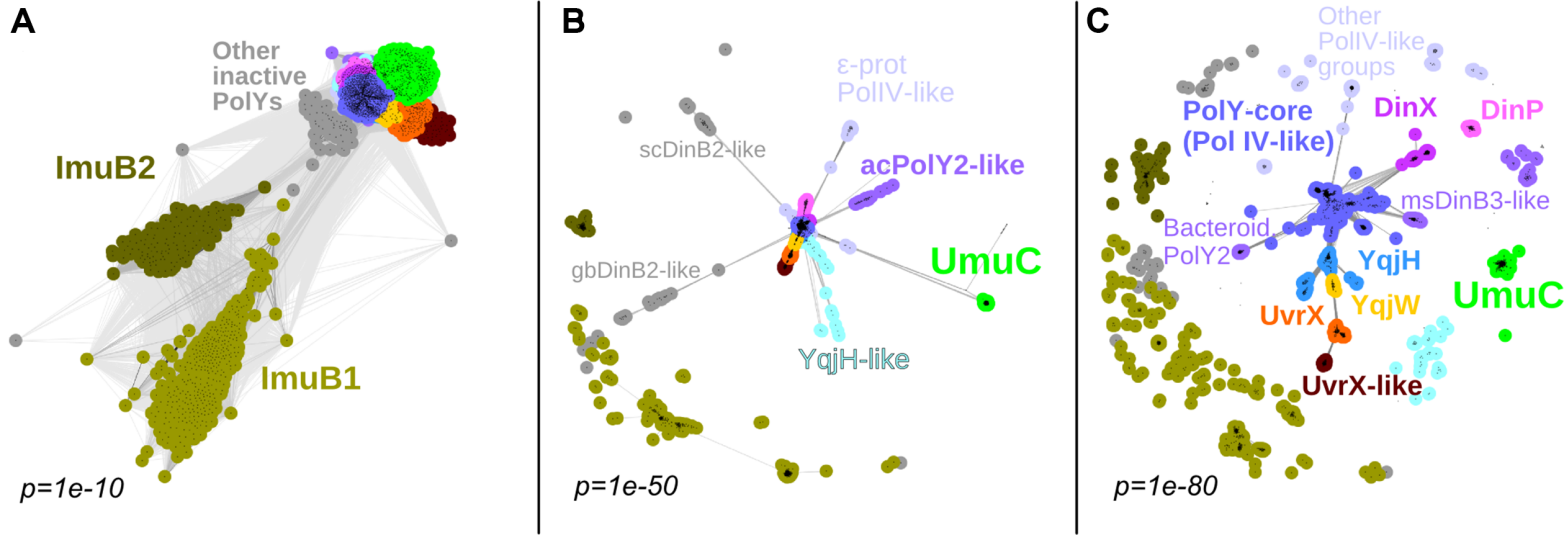
Clustering series of full-length sequences of Y-family polymerases based on their pairwise similarities. Each dot represents a single sequence and connections between them represent pairwise similarities. Results (**A**–**C**) are shown at three different similarity cutoffs defined by *P*-values. The number of sequences is the same in all three cases. Discerned polymerase groups are denoted by different colors and some are labeled. ImuB1 is represented by *Pseudomonas aeruginosa* ImuB (accession: AAG04059.1); ImuB2, *Mycobacterium tuberculosis* ImuB (CCP46215.1); UmuC, *Escherichia coli* UmuC (AAC74268.1); ‘ϵ-prot PolIV-like’, *Arcobacter nitrofigilis* PolIV (ADG92494.1); ‘acPolY2-like’, *Acidobacterium capsulatum* PolY2 (ACO33562.1); YqjH-like, *Mycoplasma hominis* MucB (CAX37259.1); YqjH, YqjW and UvrX, *Bacillus subtilis* PolY1 (CAB14319.2), PolY2 (CAB14303.1) and UvrX (CAB14068.2), respectively; UvrX-like, *Bifidobacterium bifidum* PolY (ADP36361.1); DinX and DinP, *M. tuberculosis* DinX (CCP44301.1) and DinP (CCP45865.1) respectively; ‘msDinB3-like’, *Mycobacterium smegmatis* DinB3 (ABK74774.1); ‘Bacteroid PolY2’, *Beliella baltica* DinB (AFL82914.1); ‘scDinB2-like’, *Streptomyces coelicolor* DinB2 (CAB50953.1); ‘gbDinB2-like’, *Geobacter bemidjiensis* DinB2 (ACH37529.1); PolY-core, *E. coli* DinB (AAC73335.1).

To investigate the global diversity of bacterial Y-family DNA polymerases, we obtained a representative set of bacterial genomes and corresponding proteomes. In this set, we identified 2544 Y-family members including apparently inactive polymerases (Supplementary Table S2). Next, we clustered all Y-family homologs according to their pairwise sequence similarities. Depending on the stringency of clustering threshold, the number of distinct groups (subfamilies) varies. Therefore, we performed a series of clustering experiments by gradually increasing the stringency for sequence grouping. Clustering series were performed until groups known to be functionally different—such as those typified by *E. coli* DinB and UmuC, or by *B. subtilis* YqjH (PolY1) and YqjW (PolY2)—could be distinguished. The snapshots of these clustering series are presented in Figure 1 with more detailed results provided in Supplementary Figure S1. Two major observations can be made from clustering results. First, different subfamilies show highly uneven similarity to the central cluster of Y-family sequences (PolY-core) typified by DinB proteins from *E. coli* and other bacteria. Second, at the point of separation of functionally characterized Y-family groups (Figure 1C), the overall diversity of the bacterial Y-family is fairly high. This diversity clearly exceeds the canonical division of bacterial proteins into DinB, UmuC and ImuB subfamilies. ImuB proteins, lacking conserved active site residues, are the most distinct from the remaining groups. Moreover, even when other Y-family members still cluster together, ImuB splits into two distinct groups, ImuB1, mostly present in Proteobacteria (e.g. *Pseudomonas aeruginosa*) and Acidobacteria, and ImuB2, which is typical for Actinobacteria (e.g. *M. tuberculosis*). In addition to ImuB, there are other groups of inactive Y-family homologs, but they tend to show higher similarity to the PolY-core sequences. Notably, orthologs of *E. coli* UmuC also represent one of the most distinct subfamilies. UmuC homologs are present in various gram-negative bacteria phyla such as Proteobacteria, Actinobacteria, Bacteroidetes and Cyanobacteria. Other groups typified by functionally characterized DNA polymerases such as *B. subtilis* YqjH and YqjW, and *M. tuberculosis* DinB1 (DinX, encoded by Rv1537) and DinB2 (DinP, encoded by Rv3056), are more closely related to each other and to the PolY-core. The group including the prophage-encoded *B. subtilis* UvrX and YozK-YobH is most similar to the YqjW group, but more distant from the YqjH and PolY-core groups. YqjH, YqjW and UvrX groups were identified only in gram-positive bacteria, primarily in Firmicutes, whereas a closely related UvrX-like group was found in various bacteria (Actinobacteria, Bacteroidetes, Proteobacteria, Firmicutes). Several groups (DinP, DinX, msDinB3-like) were found predominantly in Actinobacteria. Notably, polymerases of the central PolY-core group were observed to be scattered throughout the entire bacterial kingdom. To substantiate the clustering results, we also performed a phylogenetic analysis from which we excluded ImuB proteins, other inactive members of Y-family and several small groups as too diverged. The phylogenetic tree constructed for the remaining groups showed good overall agreement with clustering results, especially for major polymerase groups (Supplementary Figure S2).

### Multiple Y-family groups feature C-terminal extensions that share common motif with UmuC

To further explore the observed high diversity of Y-family members, we analyzed their domain compositions. To this end, we constructed multiple sequence alignments for each group and examined whether there were any other domains present beyond the region common for the Y-family. Some groups, exemplified by *M. tuberculosis* DinP, have only the polymerase region. Others, similarly to the *E. coli* DinB, additionally have the β-clamp binding motif at the C-terminus. A large number of groups have C-terminal extensions following the common Y-family region and the β-clamp binding motif (if present). This category comprises different groups represented, for example, by *E. coli* UmuC, *B. subtilis* polymerases YqjH, YqjW and UvrX, and *M. tuberculosis* ImuB.

**Figure 2.**
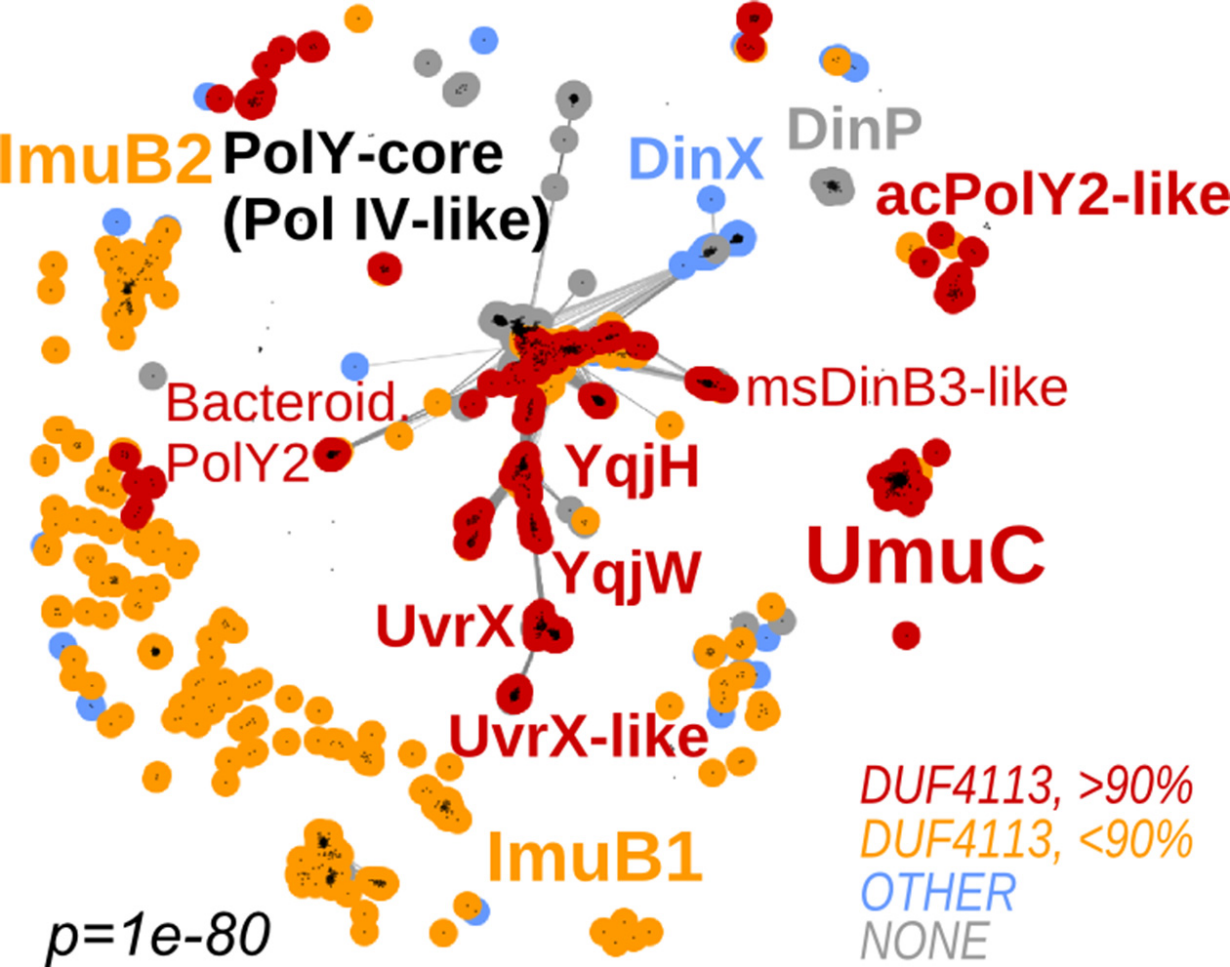
Distribution of DUF4113-containing C-terminal extensions among Y-family polymerases. Clustering snapshot is the same as in Figure 1C. Group labeling corresponds to Figure 1. Sequences are colored based on DUF4113 detection results. Red and orange colors indicate highly confident and less confident match to DUF4113, respectively; blue color indicates sequences having C-terminal regions without DUF4113; gray color indicates sequences that do not have additional C-terminal extensions.

**Figure 3.**
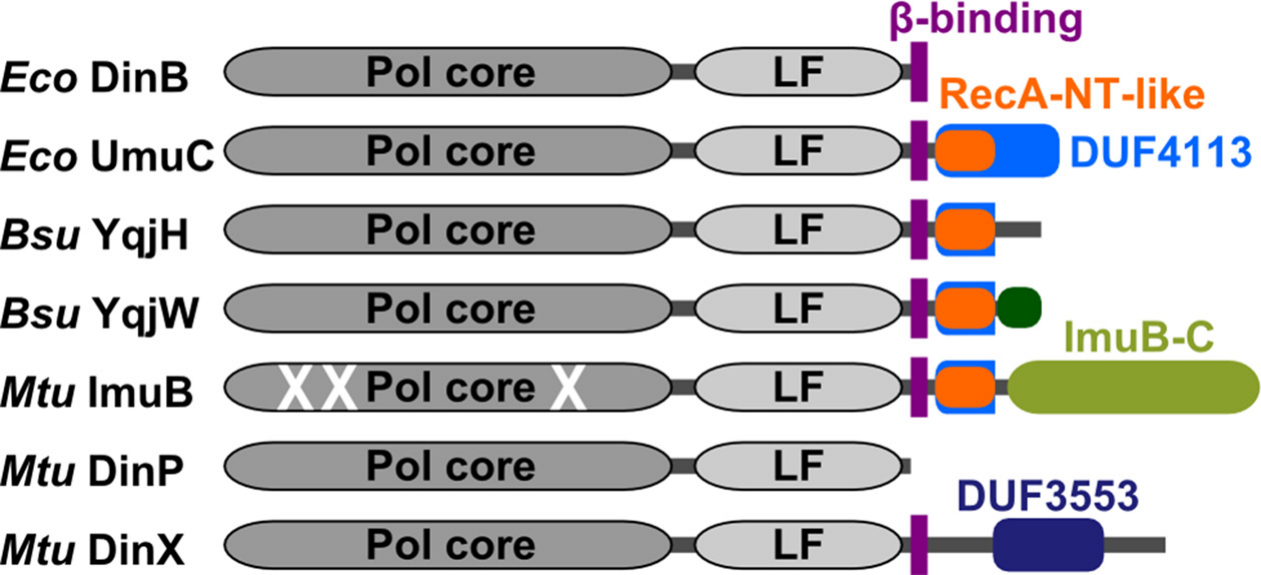
Schematic representation of domain architectures of representative Y-family DNA polymerases (*Eco, E. coli*; *Bsu, B. subtilis*; *Mtu, M. tuberculosis*). *Bsu* UvrX has identical domain composition as YqjW, their conserved C-terminal motif is denoted as a dark green shape.

To investigate whether any of these C-terminal extensions represent known structural or functional domains/motifs, we took only the C-terminal sequence regions of all Y-family members and, for each of them, performed sensitive homology searches using HHpred. The C-terminal extension of UmuC is classified in the Pfam database as a member of DUF4113 (domain of unknown function; Pfam id: PF13438). To our surprise, a match to DUF4113 was present in all groups with C-terminal extensions, except that typified by *M. tuberculosis* DinX (Figure 2 and Supplementary Figure S3). In the latter case, the C-terminal extension matched another uncharacterized Pfam domain, DUF3553 (Pfam id: PF12073). Most DUF4113 matches were highly significant (HHsearch probabilities over 90% for majority of sequences in each group). Only matches in ImuB sequences showed lower probabilities. To ensure that the DUF4113 match in ImuB sequences was not a random match, we performed a reciprocal search, a standard approach to substantiate or refute sequence homology (58,59). Using a DUF4113 sequence alignment (constructed from sequences obtained from the Pfam database) as a query, we performed searches against profile HMMs of *M. tuberculosis* and *P. aeruginosa* proteomes. In both cases, DUF4113 produced significant matches (∼90% probability) to the corresponding ImuB proteins. Taken together, these results indicate that the C-terminal extensions of the majority of Y-family groups share homology with the UmuC C-terminal extension (DUF4113). Notably, the homologous region comprises only the N-terminal part of DUF4113 (∼30 residues), apparently too short to form a globular domain, but sufficient to constitute a conserved structural motif (Figure 3).

### Common motif of C-tails in PolYs is homologous to the RecA oligomerization motif

**Figure 4.**
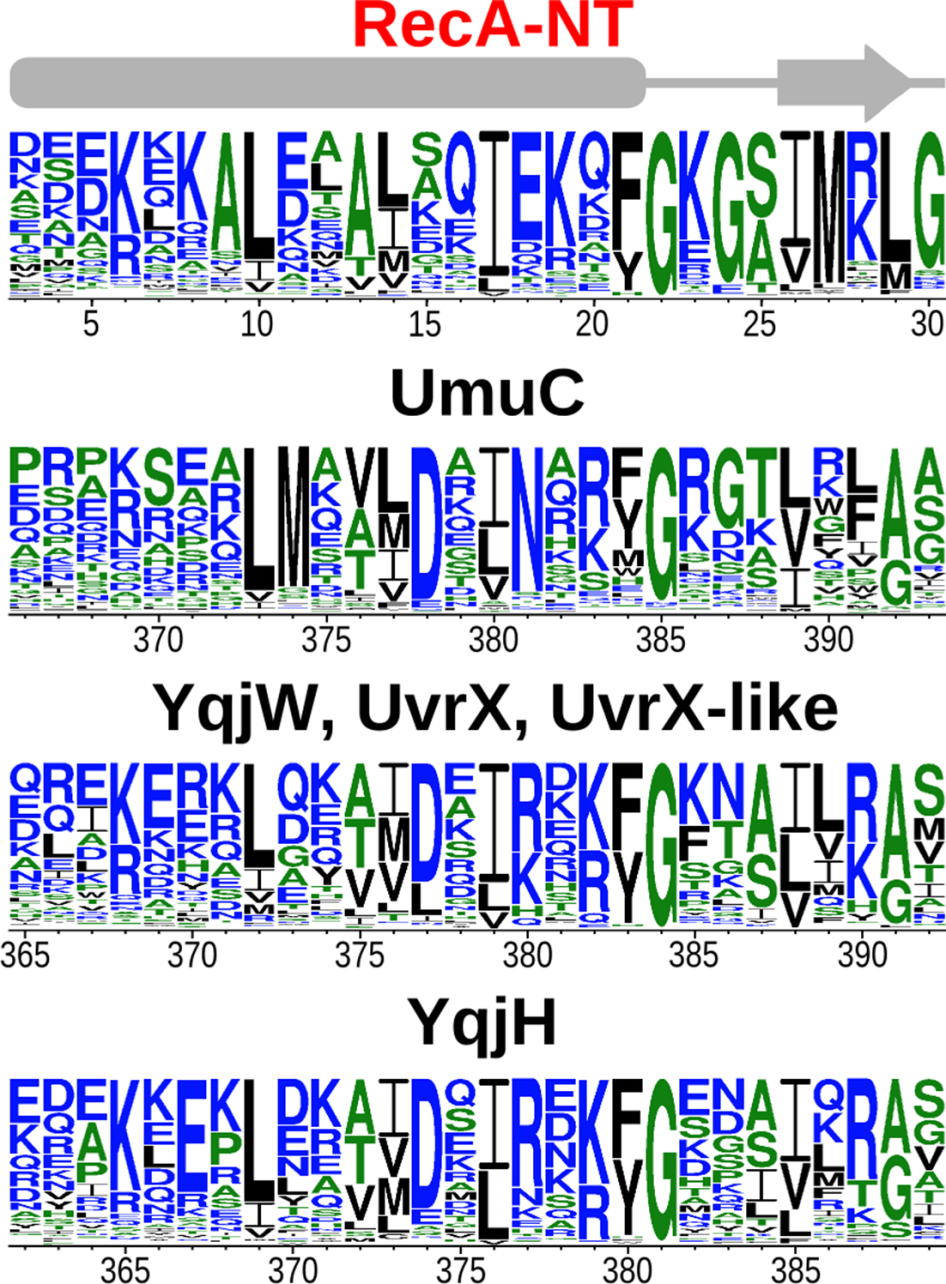
Sequence logos for the RecA-NT motif and corresponding motifs of UmuC (*Escherichia coli*) and groups with representatives in *Bacillus subtilis*. Highly similar motifs of YqjW, UvrX and UvrX-like polymerases were merged into a single logo. Secondary structure (α-β) for RecA-NT is depicted above.

To explore this common motif further, we initiated HHpred searches against protein structures in PDB, using every C-terminal extension sharing homology with DUF4113. These searches failed to detect any highly significant matches. Intriguingly, however, among the lower scoring matches (HHsearch probabilities < 68%) we identified the N-terminal region of RecA, known to mediate RecA oligomerization (60,61). We considered the match to RecA to be of particular interest because RecA is a component of active Pol V (7). RecA is also required for *B. subtilis* YqjW function (19). Therefore, we decided to explore this putative homology in detail. When we repeated the same searches using just the common motif of C-terminal extensions, the matches to RecA improved considerably. However, even the best-scoring matches to RecA obtained with *B. subtilis* YqjW and UvrX reached HHpred probabilities just above 80%. To further investigate whether this match represents true homology or is just a spurious assignment, we again performed a reverse search starting with RecA. For this, we retrieved Pfam sequences assigned to the RecA family (Pfam ID: PF00154), realigned them and used the N-terminal region of the alignment (Supplementary File S1) encompassing 40 residues of *E. coli* RecA to search Pfam profile HMMs. This search retrieved DUF4113 as a highly significant match (94% HHsearch probability). When we used an alignment of full-length RecA sequences instead of the N-terminal fragment, we were still able to find DUF4113 in the list of matches, but with lower probability. Importantly, the alignment was confined to the same motifs in both DUF4113 and RecA. This motif corresponds to the N-terminal α-helix and β-strand of *E. coli* RecA (RecA-NT). The similarity between conservation patterns in the RecA-NT motif and in different groups of Y-family polymerases is striking, in particular for those positions corresponding to the C-terminal half of RecA α-helix and the glycine at the end of the helix (Figure 4 and Supplementary Figure S4). To scrutinize this putative homology further, we performed yet another computational experiment. We collected bacterial RecA sequences and their homologs including bacterial RadA (62), ImuA (31), SulA (63) and KaiC (64). We then retained only their N-terminal fragments preceding the conserved ATPase core and clustered these fragments together with C-terminal regions of Y-family groups containing the DUF4113 motif. The results showed that the N-terminal motifs of RecA proteins, but not of their homologs, cluster together with C-terminal extensions of Y-family members (Supplementary Figure S5). Thus, multiple lines of evidence strongly suggest that the matching motifs in the N-terminus of RecA and C-terminal regions of Y-family polymerases are truly homologous.

### The oligomeric structure of RecA implies an analogous interaction mode between UmuC and RecA

**Figure 5.**
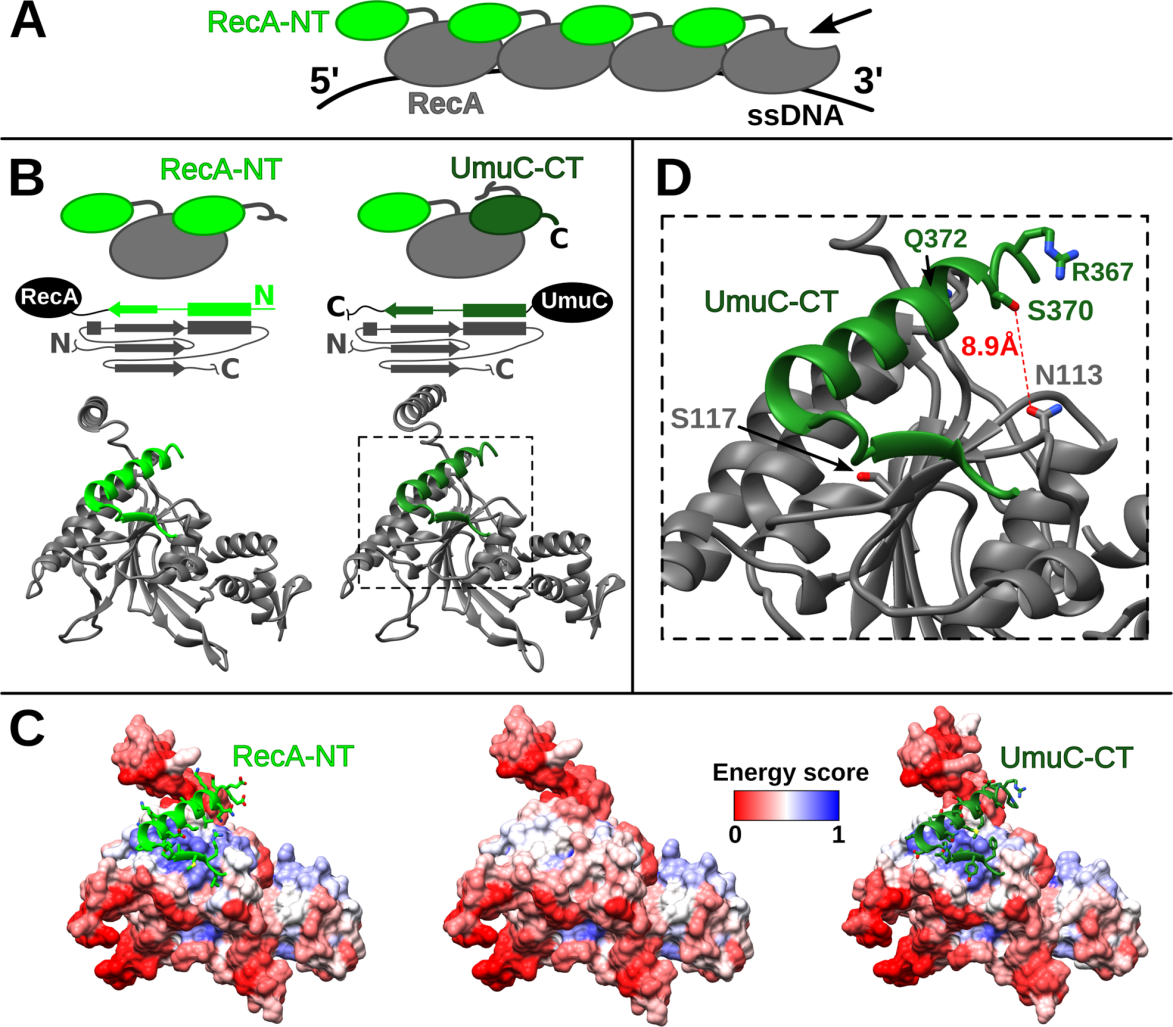
Comparison of *Escherichia coli* RecA-NT and UmuC-CT complexes with RecA. (**A**) Schematic representation of RecA filament on ssDNA. Arrow indicates the only free interaction site on the RecA surface at the 3′-end of RecA filament. (**B**) Schematic and structural comparison of the two complexes. (**C**) Interface energetic scores depicted as colored surfaces for RecA bound to RecA-NT (left), not bound to any motif (middle) and bound to UmuC-CT (right). Blue and red colors correspond to estimated favorable-unfavorable surface energy. (**D**) Zoomed-in interaction interface between *E. coli* UmuC-CT and RecA. The corresponding part of the structure is indicated with a dashed line in part (B). UmuC-CT residues S370, R367 and Q372 that have been shown to crosslink to the RecA position 113 are highlighted. The RecA S117 residue located at the interface is also highlighted.

The active form of *E. coli* Pol V (UmuD'_2_C-RecA-ATP) contains a single RecA subunit that interacts directly with UmuC (7,15). The RecA-NT motif mediates the interaction with the neighboring RecA protomer in a RecA filament (60). This immediately suggested to us that the homologous motif within the C-terminal tail of UmuC may bind RecA in the same manner (Figure 5A and B). To test whether the proposed binding mode was feasible, we generated a homology model of the RecA-NT-like fragment of UmuC bound to RecA (Supplementary File S2). Visual inspection of the modeled complex confirmed that the interface was very similar to the RecA–RecA interface defined by the oligomerization motif (Figure 5B). Moreover, the energy estimation suggested that, similarly to the RecA–RecA interaction, the UmuC–RecA interaction is energetically favorable (Figure 5C).

We next asked whether the model for the UmuC–RecA interaction was consistent with experimental findings. A recent study reported that Pol V interacts with the RecA surface defined by residues 112–117 (15). Notably, the same RecA surface is at or near the subunit–subunit interface formed by the oligomerization motif within a RecA filament (60). Moreover, when a residue of p-benzoyl-phenylalanine (Bpa) was substituted for Asn at position 113 in RecA, it could be readily crosslinked to UmuC (15). One of the two UmuC fragments involved in crosslinking was independent of the Pol V activation state and involved residues 361–376. Within this fragment, Ser 370 was identified as the most frequently crosslinked residue (15). In the structural model of the RecA–UmuC interaction, side chains of RecA Asn 113 and UmuC Ser 370 are closer than 10 Å and are positioned such that Bpa at position 113 could be very close or in direct contact with Ser 370 (Figure 5D). Two other UmuC residues (Arg 367 and Gln 372) that were also observed to crosslink to Bpa 113 showed significantly lower crosslinking efficiency. In the model, these two residues are in close proximity to Ser 370, but the orientation of their side chains is less favorable. Therefore, the structural model in which UmuC interacts with RecA in the same way as RecA monomers interact with each other in a RecA filament is entirely consistent with the experimental data. The model also explains why the assembly of the active form of Pol V requires recruitment of the RecA subunit specifically from the 3′-end of RecA filament (7). It is apparent that only the RecA subunit at the 3′-end has its oligomerization surface exposed and thus available for UmuC binding (Figure 5A).

### The conserved interaction between Y-family members and RecA or ImuA may be ubiquitous

To our knowledge, except for *E. coli*, direct binding of RecA to a Y-family DNA polymerase has not been demonstrated. On the other hand, the widespread presence of the conserved RecA-NT-like motif in Y-family (Figure 4 and Supplementary Figure S4), coupled with the experimentally supported structural model of the UmuC–RecA complex, suggests that such a conserved interaction may be ubiquitous. To test this idea, we generated structural models of corresponding complexes for *B. subtilis* Y-family polymerases YqjH, YqjW and UvrX, all of which possess a RecA-NT-like motif. Both manual assessment and energy estimation revealed that, in all cases, models represent favorable interactions (Supplementary Figure S6) consistent with high sequence conservation of the motif. The conservation can also be observed at the level of residue–residue interactions. For example, in the crystal structure of *E. coli* RecA, Lys 6 of one monomer forms a salt bridge with Asp 139 of another monomer. The corresponding salt bridge is retained in modeled complexes of *B. subtilis* Y-family polymerases with RecA (Supplementary Figure S7). Moreover, many UmuC homologs maintain a positively charged residue in the position corresponding to Lys 6 of *E. coli* RecA (Figure 4, position 369 of UmuC).

In addition to active (known or putative) Y-family DNA polymerases, we also identified the RecA-NT-like motif in ImuB proteins. *Mycobacterium tuberculosis* ImuB is essential for induced mutagenesis by DnaE2, and its C-terminal region mediates interaction with both ImuA and DnaE2 (31). Since ImuA is a RecA homolog, we considered that the interaction between ImuB and ImuA may also be analogous to the RecA–RecA interaction. Consistent with this prediction, a structural model of the complex between ImuB and ImuA did not have any obvious flaws. Intriguingly, a structural model in which the same ImuB motif was complexed with RecA also appeared feasible and, in fact, more energetically favorable (Supplementary Figure S6). To date, no experiments probing the ImuB interaction with RecA have been reported but, based on our analysis, such an interaction appears plausible.

### YolD homologs appear to be UmuD counterparts in gram-positive bacteria

So far, UmuD proteins have not been detected in gram-positive bacteria, but proteins of the YolD family (Pfam ID: PF08863) have been proposed as *E. coli* UmuD analogs (17). It was shown that *B. subtilis* YqjX (a YolD homolog) together with YqjW is induced in the SOS response (21), mirroring the behavior of UmuD and UmuC in *E. coli*. However, experiments addressing the role of YqjX in YqjW-mediated mutagenesis were inconclusive (19). Therefore, we decided to comprehensively reassess the putative functional link between Y-family polymerases and YolD homologs.

**Figure 6.**
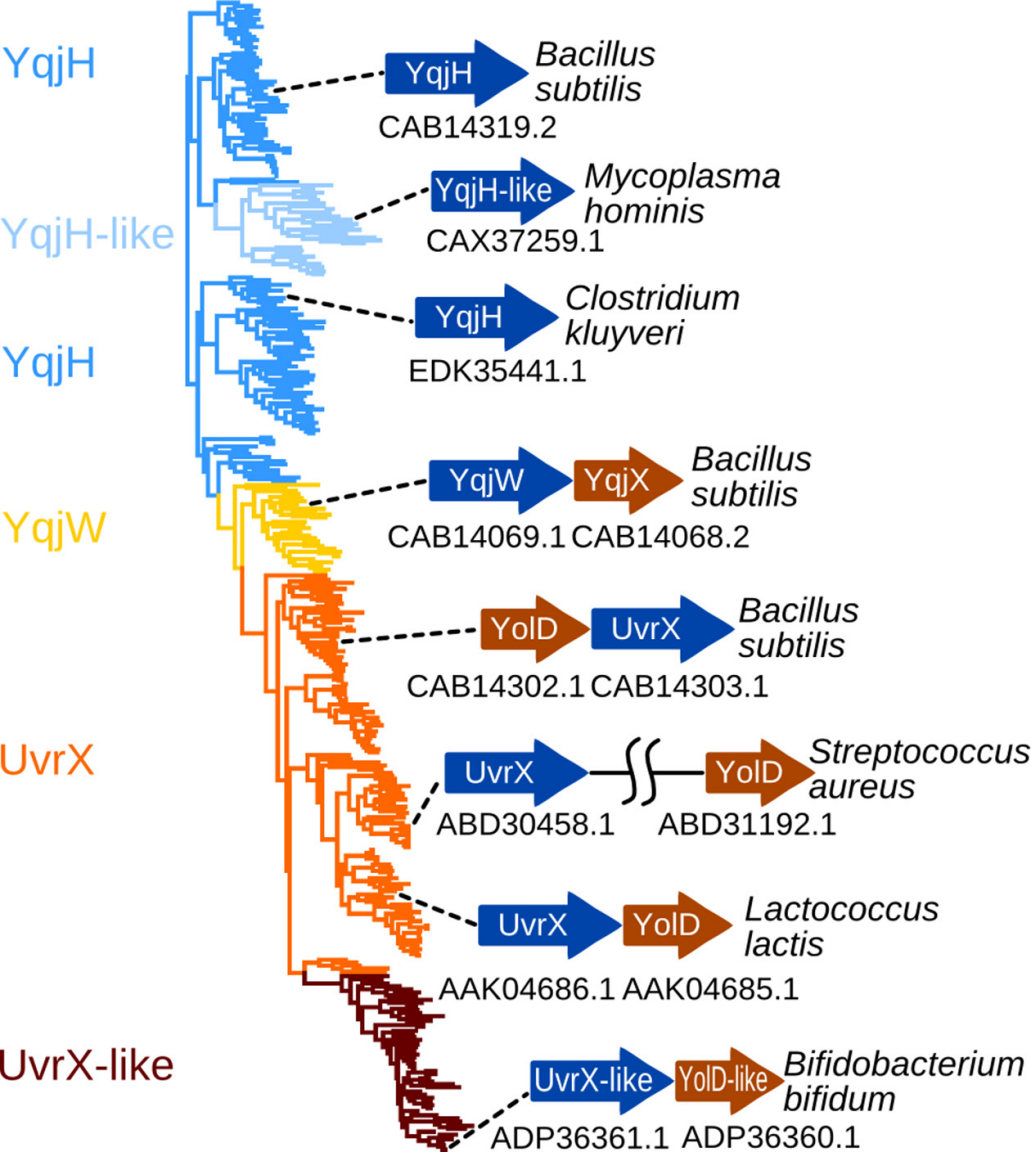
Phylogenetic tree of YqjH(-like), YqjW and UvrX(-like) sequences. Typical operons of selected representatives are shown with corresponding accession numbers.

First, we asked how typical is the presence of YolD family proteins in genomes encoding YqjW, UvrX or UvrX-like proteins. It turned out that the co-occurrence is nearly perfect with only three exceptions among 207 cases. Moreover, the majority of *yolD* homologs were located in the immediate vicinity of genes encoding YqjW, UvrX or UvrX-like proteins, suggesting that they belong to the same operon and therefore are functionally linked (Figure 6; Supplementary Figure S8 and Supplementary Table S3). In a number of cases, especially in Staphylococci, a YolD homolog is encoded at a distance from a Y-family polymerase. However, in *Staphylococcus aureus*, it was shown that, despite being in different genome regions, both *uvrX* (SACOL1400) and y*olD* (SACOL1986) homologs have LexA-binding sites and both are upregulated in the SOS response (65). In contrast, we did not observe enrichment of *yqjH* and *yqjH*-like genomic neighborhoods with *yolD* homologs, suggesting that YqjH and YolD homologs are not functionally linked.

**Figure 7.**
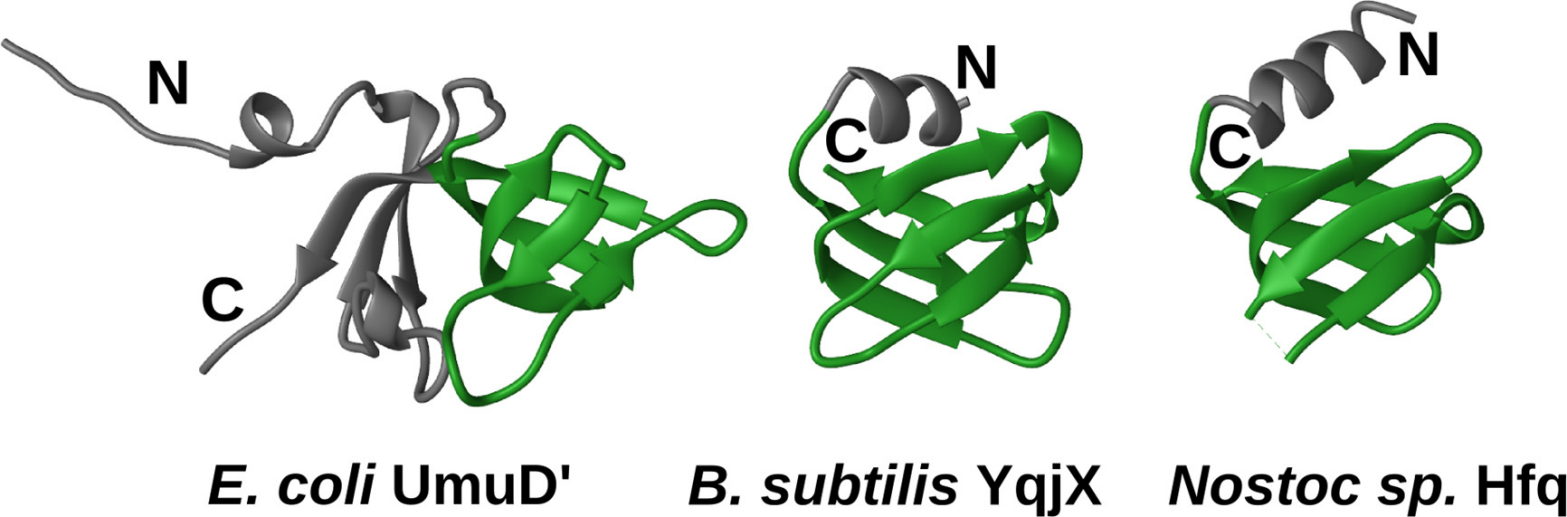
Comparison of *Escherichia coli* UmuD′ (left, PDB ID: 1AY9), *Bacillus subtilis* YolD model (middle) and cyanobacterial (*Nostoc sp*.) Hfq protein (right, PDB ID: 3HFN). Common structural parts are colored green.

Next, we focused on structural features of the YolD family, for which no solved structures are available. Nonetheless, the YolD family has been previously shown to be part of the WYL-like superfamily, predicted to have an SH3 β-barrel fold related to Sm domains (66). Our searches using HHpred and *B. subtilis* YqjX and YolD as queries against PDB corroborated this previous prediction. Among the closest detected structural matches were *E. coli* YaeO, a Rho-specific inhibitor of transcription termination (67), and a cyanobacterial Hfq homolog (68). Based on detected similarities, we constructed homology models for YqjX and YolD (Supplementary Files S3 and S4). Model assessment showed that YqjX and YolD sequences are indeed compatible with the SH3 β-barrel structure (Supplementary Figure S9). Interestingly, UmuD' has an all β-structure (69) that also encompasses an SH3 β-barrel as part of its fold. Thus, YolD/YqjX and UmuD' have structurally similar cores (Figure 7), but represent evolutionarily distinct families as sequence-based searches failed to detect this structural similarity.

Finally, we looked for potential YolD-binding sites. UmuC has a conserved region of about 28 residues at the very C-terminus, that was shown to represent a major UmuD'-binding site (13,14). Our analysis revealed that proteins in the YqjW, UvrX and UvrX-like subfamilies also have a conserved C-terminal tail and the conservation patterns are very similar in all three subfamilies (Supplementary Figure S10). This observation suggests that, similarly to Pol V, the conserved tails of YqjW, UvrX and UvrX-like proteins may serve as binding sites for YolD homologs. Notably, in YqjH homologs that are not associated with the YolD family, C-terminal tails are generally variable or missing altogether.

Collectively, our results revealed strong parallels between YolD and UmuD families: both have structures with SH3 β-structural cores, and both are associated with polymerases that have RecA-NT-like motifs followed by conserved C-terminal tails. Therefore, while UmuD' is known to do so, YolD is expected to bind the corresponding conserved C-terminal tail.

### Modules of the ImuA-ImuB-DnaE2 system are present in other putative mutasomes

**Figure 8.**
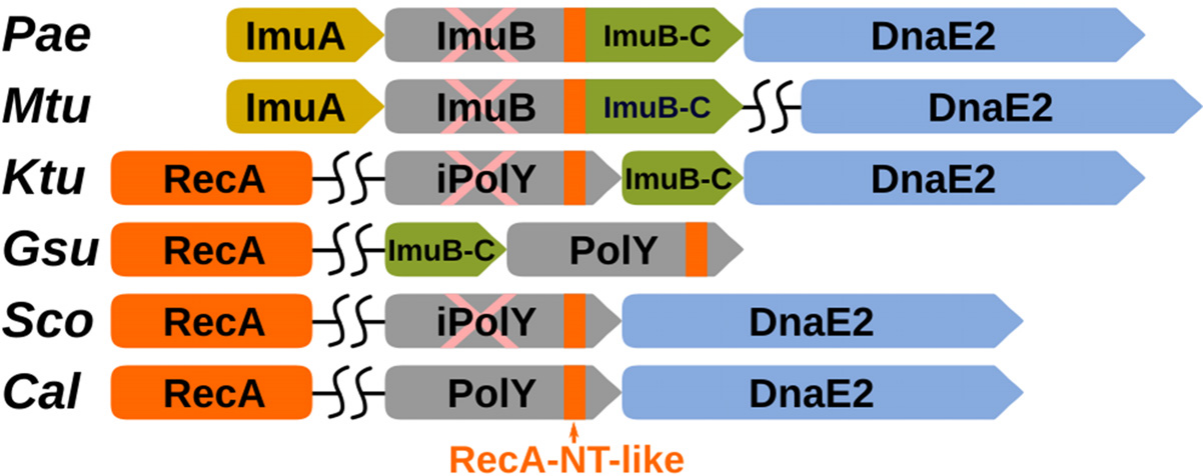
Typical PolY operons with DnaE2 and/or ImuB-C in representative organisms (corresponding PolY accession ID indicated in parentheses): *Pae, Pseudomonas aeruginosa* (AAG04059.1); *Mtu, Mycobacterium tuberculosis* (CCP46215.1); *Ktu, Kyrpidia tusciae* (ADG05750.1); *Gsu, Geobacter sulfurreducens* (AAR33378.1); *Sco, Streptomyces coelicolor* (CAB50953.1); *Cal, Cellulophaga algicola* (ADV50500.1). RecA-NT-like motifs of PolYs are indicated with orange rectangles. Inactive polymerases are marked with pink ‘X’.

ImuB proteins also have a conserved C-terminal region (ImuB-C) following the RecA-NT-like motif (Figure 3). ImuB-C is significantly longer compared to the C-terminal tails of either UmuC or YqjW/UvrX/UvrX-like. A previous study has found that ImuB-C represents a conserved domain that may also be encoded as a standalone protein (32). Prompted by this observation, we sought to investigate whether there are systems similar to ImuA-ImuB-DnaE2, but with a standalone ImuB-C. We explored gene neighborhoods of various Y-family groups other than ImuB and found that members of some of these groups indeed form putative operons with *imuB-C* and/or *dnaE2* (Figure 8 and Supplementary Table S4). Often, but not universally, these operons also include *lexA*. Notably, we have not found *imuA* to be present either in these putative operons or in the entire genomes. However, both active and inactive Y-family members in these groups do have the RecA-NT-like motif suggesting that they may bind RecA instead of ImuA.

A group of diverse inactive Y-family polymerases, represented by the one from *Kyrpidia tusciae*, were identified in putative operons having the most similar composition to that of *imuA-imuB-dnaE2*. The operon includes genes of an inactive Y-family polymerase, a standalone ImuB-C and DnaE2. RecA presumably may replace the missing ImuA.

Another putative ImuA-ImuB-DnaE2 variant is represented by an apparently active Y-family polymerase coupled with a standalone ImuB-C. We identified one such group of polymerases (GeoPolY-like, Supplementary Figure S2) belonging to PolY-core in δ-proteobacteria, Deferribacteres and Planctomycetes. The evidence that such a system represents a functional unit were provided in *Geobacter sulfurreducens*. The *lexA-imuB-C-polY* operon in this gram-negative δ-proteobacterium was shown to be induced in response to DNA damage and to be transcribed as a single unit (70). In addition, we observed coupling of ImuB-C with two subgroups of YqjH polymerases originating mostly from Clostridia. Thus, in these cases, a putative multisubunit complex might be composed of a Y-family polymerase, RecA and ImuB-C. The presence of catalytically active Y-family polymerase presumably removes the necessity for another active polymerase, explaining the absence of DnaE2-coding gene in these operons.

We also identified two putative systems, in which a Y-family member is coupled with DnaE2, but ImuB-C is missing. The first is typified by one of the two Y-family members (DinB2) and DnaE2 in *Streptomyces coelicolor* (71). According to our analysis, *S. coelicolor* DinB2 is an inactive Y-family polymerase related to the PolY-core sequences (Figure 1B, scDinB2-like group). The second putative system identified in Bacteroidetes is unusual, because it involves two apparently active polymerases, a Y-family polymerase belonging to Bacteroidetes PolY2 group and DnaE2 (represented by the *Cellulophaga algicola* system in Figure 8). Thus, the presumed composition of the mutasome in these two cases would include active or inactive Y-family polymerase, RecA and DnaE2.

Among structural–functional modules that make up the ImuA-ImuB-DnaE2 system and its putative variants identified here, ImuB-C is characterized least. We failed to find any structures or functional domains related to ImuB-C; however, secondary structure prediction indicates predominantly β-structure. Previously, Aravind *et al.* also noticed the β-structural character of this region and even ventured to propose that it might represent an SH3-like β-barrel domain (32). Although we were unable to substantiate this proposition, a β-barrel-like structure seems plausible, and in the context of ImuB the ImuB-C domain appears to be the primary candidate for DnaE2 binding.

### DinX features protein-interacting Tudor domain

**Figure 9.**
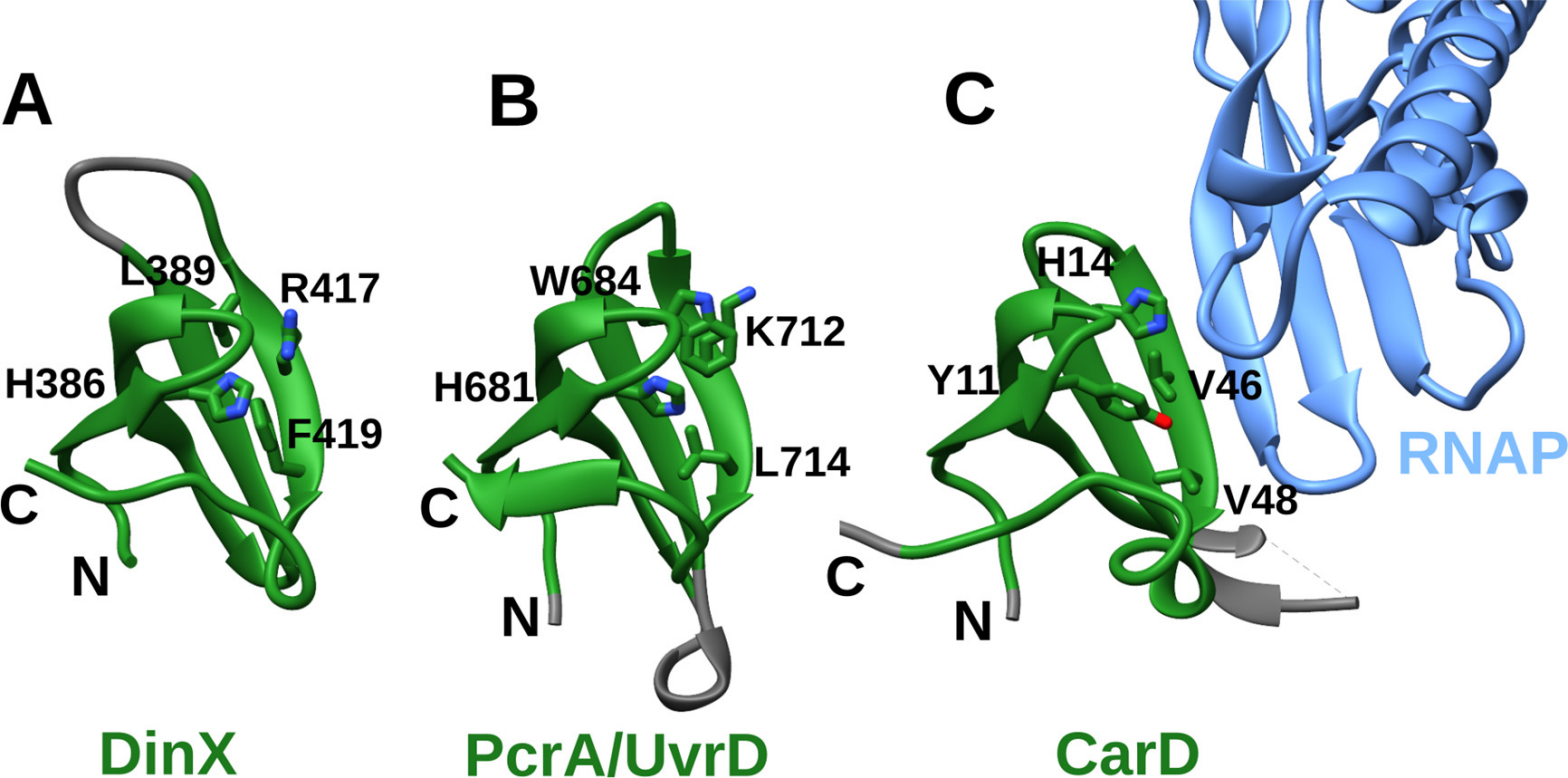
Comparison of Tudor domains from three proteins: (**A**) Modeled Tudor-like domain of *Mycobacterium tuberculosis* DinX; (**B**) Tudor domain of *Geobacillus stearothermophilus* PcrA/UvrD helicase (PDB ID: 5DMA); (**C**) Tudor domain of CarD bound to RNAP β subunit (light blue) (PDB ID: 4KBM). Common parts are highlighted in green. Conserved residues determined to be important for PcrA/UvrD binding to RNAP are labeled and their side chains are shown. Side chains of residues in corresponding positions of other structures are also shown.

Our investigation into the C-terminal extensions of Y-family groups (see above) revealed that the C-terminus of *M. tuberculosis* DinX is different from the rest. It does not contain a RecA-NT-like motif but, instead, features a domain of the DUF3553 family surrounded by regions of predicted intrinsic disorder. Since this was the only other prominent domain and it was confined to the DinX group, we sought to find out more about its possible structure and function. Homology searches against PDB identified it as a Tudor-like domain, the closest structural homolog being the RNA polymerase interacting domain at the C-terminus of PcrA/UvrD helicase (72). Homology modeling confirmed that the C-terminal domains of DinX and PcrA/UvrD are closely related (Figure 9;Supplementary Figure S9 and Supplementary File S5). Notably, the Tudor domain also adopts an SH3 β-barrel fold, but represents an evolutionarily different branch from YolD. Sanders *et al.* have compared the PcrA/UvrD Tudor domain to other Tudor domains crystallized in complexes with RNA polymerase (e.g. Tudor domain of CarD (73), shown in Figure 9C), highlighting a conserved interaction surface and identifying at least four residues, mutations of which abolish the interaction with RNA polymerase. Similar conserved residues can also be observed in the Tudor-like domain of DinX. Consequently, the similarity to the C-terminal domain of PcrA/UvrD helicase suggests that DinX may also interact with the RNA polymerase and play a role in transcription-coupled DNA repair.

## DISCUSSION

Bacterial Y-family DNA polymerases have been classified into DinB and UmuC branches, the latter further subdivided into gram-negative and gram-positive subgroups (3). While generally agreeing with this broad division, our results show that there are many more distinct groups, some of them with no experimentally characterized representatives. The global diversity of Y-family is considerable. In particular, ImuB proteins and other apparently inactive polymerases display greatly increased divergence from the active Y-family DNA polymerases. This is entirely consistent with the observation that the presence of an active site induces strong evolutionary constraints on a large fraction of residues in a protein (74). Once the active site is lost, the selection pressure is relieved, allowing protein sequences to evolve faster. Another important contributor to the observed diversity of the Y-family polymerases is the variety of C-terminal regions that are present in some subfamilies and extend beyond the common structural core of the family. None of these C-terminal regions have been structurally characterized and data on their functional role(s) remain scarce. The roles of C-terminal tails in representatives of only two groups, UmuC and ImuB, have been investigated to any extent.

Given the substantial diversity of Y-family groups, our finding that most Y-family groups possessing C-terminal tails share a homologous sequence motif of about 30 residues was entirely unexpected. Even more surprising was the finding that the sequence of this short motif is related to the RecA N-terminal α-β motif, involved in RecA filament formation. Homologous protein complexes are often conserved at the level of 3D structures. Therefore, this finding immediately suggested that the motif present in various Y-family polymerases may bind RecA in the same manner as RecA monomers bind each other in a RecA filament (60). Indeed, the assessment of structural models for UmuC and several other representatives suggested that such a binding mode between Y-family members and RecA is structurally feasible and energetically favorable. We found similar interaction complementarity between the modeled structures of ImuB and ImuA. ImuA is a RecA homolog lacking the conserved ATPase site (Walker A and B motifs), the C-terminal subdomain and apparently unable to self-associate (31). This suggests that ImuA may play structural role analogous to that of the RecA subunit in Pol V. Notably, the active site of RecA ATPase, essential for homologous recombination, is not needed for Pol V activity (9). Thus, it is likely that ImuA has evolved from RecA by losing the sites required for its recombination function, but not needed for its regulatory role of a TLS polymerase.

Although surprising at first, the finding that Y-family polymerases and RecA share a homologous sequence region makes perfect biological sense. Y-family polymerases are often upregulated during SOS response and are recruited to the same regions of damaged DNA as RecA. On one hand, binding to the oligomer-forming surface of RecA may be used to shift the balance between the formation of a RecA filament (facilitating homologous recombination) and a TLS complex containing RecA subunit (leading to the mutagenic DNA repair). On the other hand, binding to RecA might represent a way to regulate the activity of a Y-family DNA polymerase and/or guide the polymerase to the site of DNA damage. In *E. coli*, it has long been known that there is a competition between RecA-mediated homologous recombination and Pol V-mediated mutagenesis (75). It has been unambiguously shown that the transfer of a RecA monomer—specifically from the 3′-proximal tip of an activated RecA filament—is critical for Pol V activity, although there are opposing views regarding the guiding role of RecA (7,76). In other cases, only the RecA requirement for the function of Y-family DNA polymerases has been investigated, but not direct RecA binding. For example, it was shown in *B. subtilis* that the presence of RecA is required for untargeted mutagenesis by YqjW, but not by YqjH (19). This observation seems to be at odds with our finding that both YqjW and YqjH have strongly conserved RecA-NT-like motifs and binding to RecA for both appears energetically favorable. However, these seemingly contradictory conclusions might be easily reconciled, were RecA not always a positive regulator of polymerase mutagenic activity. Such an effect has indeed been observed at least in one case. The formation of Pol IV complex with RecA and UmuD was found to suppress the −1 frameshift mutagenesis by Pol IV and to promote accurate synthesis of DNA (16). Additional accessory subunits, as predicted in the case of YqjW (see discussion below), may also affect the polymerase activity.

In several subfamilies, the RecA-NT-like motif is followed by a conserved C-terminal tail that could serve as an additional protein-binding site. The C-tail of the Pol V UmuC subunit indeed has been found to be the primary UmuD′-binding site (13,14). Since UmuD homologs have not been identified in *B. subtilis* or other gram-positive bacteria, the possibility that these organisms possess a Pol V counterpart remained dubious. Our results provide compelling support in favor of the existence of a multisubunit mutasome. We have shown that polymerases of three closely related subfamilies (YqjW, UvrX and UvrX-like) have conserved tails and that these polymerases co-occur with YolD family proteins typically encoded in the same operon. The inferred functional link between the polymerase and a YolD homolog is supported by the observation that, in *B. subtilis* and *S. aureus*, both proteins are induced during SOS response (21,65). Moreover, we found that proteins of the YolD family share structural similarity with UmuD′ and, consequently, could play a structurally similar role. The function of *B. subtilis* YqjX (YolD homolog) along with YqjW has been investigated experimentally. However, the deletion of *yqjX* reduced UV-induced mutagenesis mediated by YqjW only about 1.5-fold relative to wild-type (19). Such a small difference may suggest that YqjX either has no role in YqjW-mediated mutagenesis or is needed only at a small fraction of lesions. Interestingly, follow-up experiments showed that over 90% of the UV-induced mutagenesis by YqjW depends on Pol I (22). However, a fraction of UV-induced mutations did not require Pol I, revealing a second pathway of mutagenesis by YqjW (22). The small effect of *yqjX* deletion is entirely consistent with the idea that this second minor pathway requires the presence of YqjX, but this has not been tested. Thus, despite ambiguity of some experimental data concerning YqjX function, multiple lines of evidence suggest that YqjW, UvrX and UvrX-like polymerases, at least in some pathways of mutagenic DNA repair, function together with a YolD homolog.

**Figure 10.**
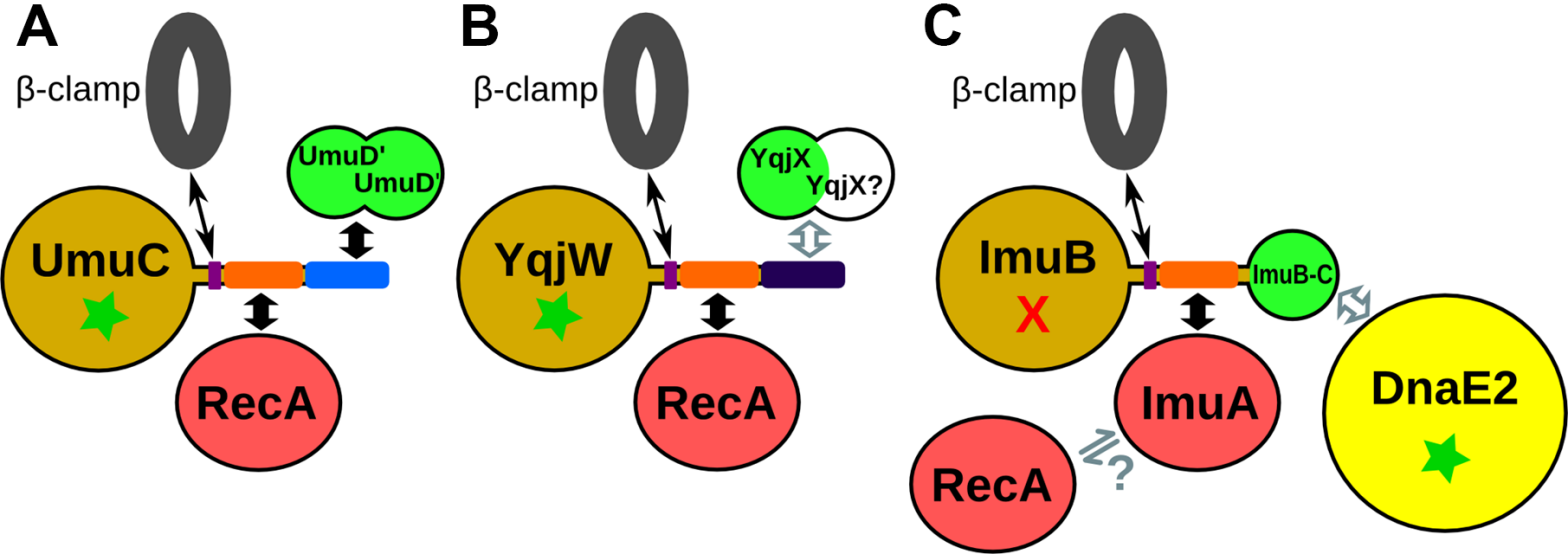
Schematic comparison of known and proposed complexes and interactions of three mutasomes: (**A**) *Escherichia coli* PolV, (**B**) *Bacillus subtilis* YqjW (also represents UvrX) and (**C**) *Mycobacterium tuberculosis* ImuA-ImuB-DnaE2. Components with similar structures are shown in the same color. Wide black arrows indicate either known interactions or interactions predicted with high confidence and supported by structural models, gray-contoured arrows indicate putative interactions without support of structural models, and narrow black arrows denote transient interactions with the β-clamp. Green star and red X indicate correspondingly the presence and the absence of the polymerase active site.

Overall, our results revealed that known or inferred multisubunit mutasomes feature modular architecture, similar in a number of ways. Figure 10 illustrates three prototypical mutasomes represented by *E. coli* Pol V, *B. subtilis* YqjW (PolY2) and *M. tuberculosis* ImuA-ImuB-DnaE2. In all three systems, the Y-family polymerase (or its inactive homolog) is an organizing subunit. This subunit has distinct regions in the C-terminal tail for interaction with the processivity factor (β-clamp) and RecA (or its inactivated homolog). Both UmuD' and YolD subunits have a common SH3-like structural core and are either known (Pol V) or expected (YqjW) to bind through the conserved C-terminal tail of Y-family polymerase. In the third mutasome, ImuB appears to represent a fusion between a Y-family homolog and an analogous small subunit (ImuB-C). Whether or not ImuB-C also represents an SH3-like β-barrel as proposed earlier (32) remains to be established, but its β-structural character is beyond doubt. Our modeling results suggest that RecA may also play a role in the function of ImuA-ImuB-DnaE2 mutasome; however, to our knowledge, no experiments addressing the ImuB–RecA interaction have been reported. In addition to the prototypical ImuA-ImuB-DnaE2 mutasome, we identified several of its putative variants that feature different combinations of individual modules. All of these putative systems lack ImuA and a typical ImuB. The latter is replaced with an active or inactive Y-family polymerase and is coupled with the standalone ImuB-C and/or DnaE2 (Figure 8). In all the variants, the ImuB replacement has a RecA-binding motif suggesting that in these systems RecA might substitute for ImuA. Although these variants are less common than the prototypical ImuA-ImuB-DnaE2 system, they offer clues how the latter might have evolved. Specifically, ImuB might have evolved by fusion of Y-family polymerase and a standalone ImuB-C protein with subsequent recruitment of DnaE2 and inactivation of the ImuB active site. ImuA might have evolved from a duplicated copy of RecA. Other variants, identified in *Streptomyces* and Bacteroidetes, suggest that there are alternative evolutionary solutions for coupling a Y-family member and DnaE2 that do not require ImuB-C.

C-terminal motifs or domains in Y-family polymerases seem to be widely used as means of regulation by providing sites of interaction with other proteins. In this regard, the subfamily typified by *M. tuberculosis* DinX is particularly intriguing. Among Y-family polymerases, it appears to be the only major subfamily featuring a C-terminal extension without the RecA-binding motif. Instead, at its C-terminus DinX has a Tudor-like domain, which has been shown to mediate the PcrA/UvrD helicase interaction with RNA polymerase (72). Biochemical characterization of the *M. smegmatis* DinX ortholog showed that it is a DNA-dependent DNA polymerase (77). However, even a wide range of *in vivo* experiments, supposed to be phenotypically revealing, failed to assign any biological function to DinX (78). The presence of the Tudor-like domain in DinX suggests that focusing experiments on transcription-coupled DNA repair might be more rewarding in search for the biological function of DinX.

As protein–protein interactions are important regulators of polymerase (e.g. Pol V) activity, they could be potential therapeutic targets. In this regard, the interaction between Y-family polymerases and RecA seems especially promising because the interaction appears to be specific to bacteria and it is coupled to the interaction between RecA monomers. In other words, by targeting the TLS synthesis (Pol Y–RecA interaction) it should be possible to simultaneously target the homologous recombination, which depends on the formation of a RecA filament (RecA–RecA interaction). In order to overcome the double-sided effect of an antagonist of this interaction, three interactors (the oligomerization motif of RecA, the polymerase RecA-NT-like motif and the RecA surface that binds either of these motifs) would have to undergo correlated changes (mutations). There has been a report of *E. coli* RecA filament assembly inhibition by rationally redesigned peptide based on RecA N-terminus (79). Although it has not been tested whether the same peptide inhibits Pol V activity, our results suggest it likely would. Such inhibitors may have the potential to be used either as stand-alone antibiotics or as ancillary agents along with existing antibacterial compounds to fight the increasing problem of antibiotic resistance.

## Supplementary Material

Supplementary Data
