# Supplementary material for: New insights into the structures and interactions of bacterial Y-family DNA polymerases
Source: Nucleic Acids Res. 2019 Mar 27;47(9):4393–405. doi: 10.1093/nar/gkz198 (PMC6511836; doi:10.1093/nar/gkz198)
Supplement: Supplementary Data [file gkz198_supplemental_files.zip › Supplementary_Figures.pdf]

New insights into the structures and interactions of bacterial Y-family DNA polymerases

SUPPLEMENTARY FIGURES

Kęstutis Timinskas and Česlovas Venclovas*

Institute of Biotechnology, Life Sciences Center, Vilnius University, Saulėtekio 7, Vilnius LT-10257, Lithuania

* To whom correspondence should be addressed.
Tel: +370-5-2234368;
Fax: +370-5-2234367;
Email: ceslovas.venclovas@bti.vu.lt

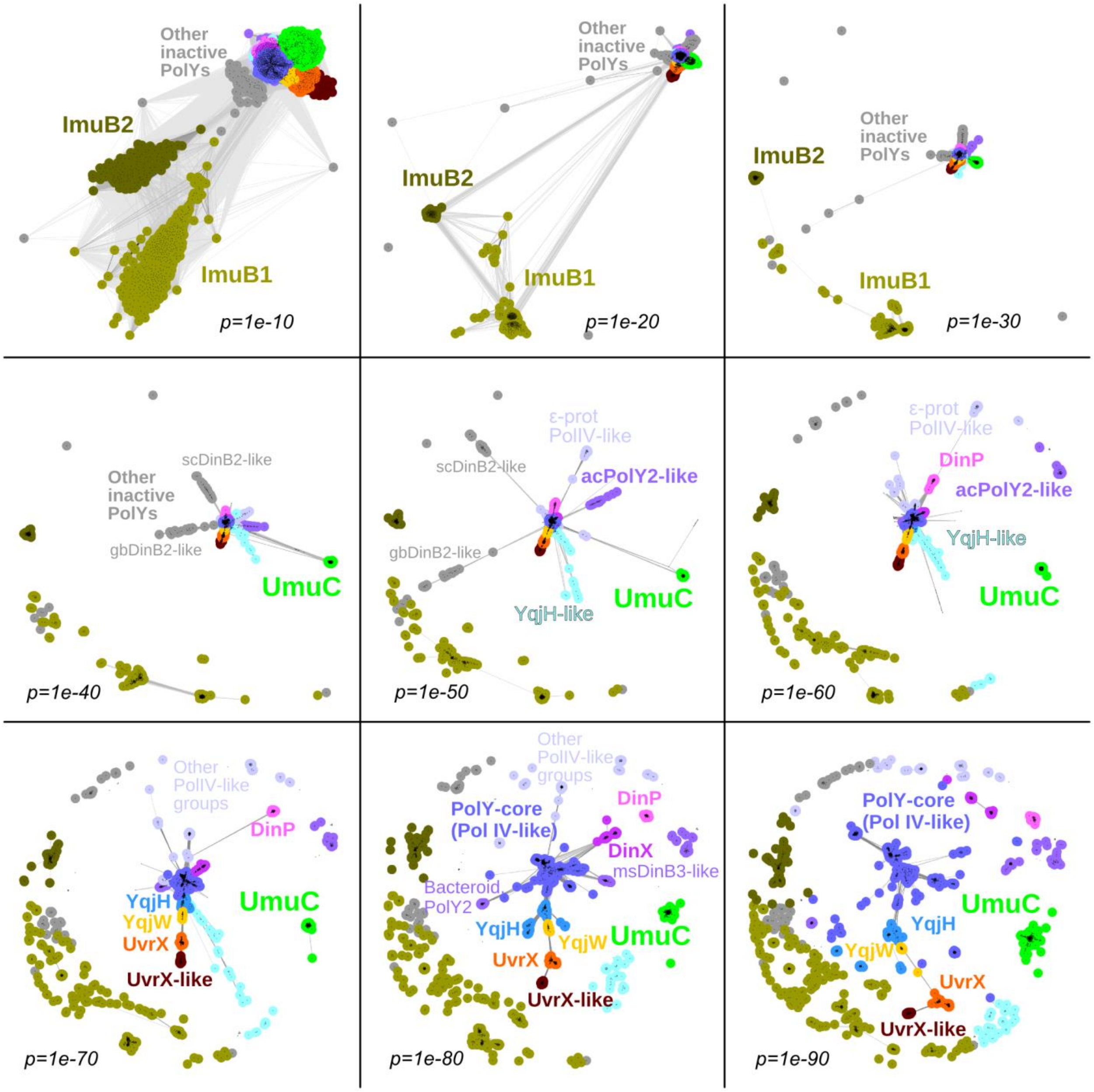

Supplementary Figure S1. Clustering series of Y family polymerase full length sequences based on their pairwise similarities: 9 snapshots at p-value cutoffs ranging from 1e-10 to 1e-90. Each dot represents one sequence and connections between them – their pairwise similarities. Discerned polymerase groups are marked by different colors and also their given names next to the group sequences. Groups with well known representatives: ImuB1 is represented by *Pseudomonas aeruginosa* ImuB (accession id AAG04059.1); ImuB2 – *Mycobacterium tuberculosis* ImuB (CCP46215.1); UmuC – *Escherichia coli* UmuC (AAC74268.1); PolY-core – *E. coli* DinB (AAC73335.1); YqjH, YqjW and UvrX – *Bacillus subtilis* PolY1 (CAB14319.2), PolY2 (CAB14303.1) and UvrX (CAB14068.2) respectively; DinX and DinP – *M. tuberculosis* DinX (CCP44301.1) and DinP (CCP45865.1) respectively. UvrX-like is similar to UvrX, but found in different phyla of bacteria (e.g., *Bifidobacterium bifidum* PolY (ADP36361.1)). YqjH-like polymerases are most similar to YqjH, but found in Tenericutes phylum of bacteria (e.g.,

Mycoplasma hominis MucB (CAX37259.1)). Polymerases of the ‘msDinB3-like’ exemplified by DinB3 polymerase of *Mycobacterium smegmatis* (ABK74774.1) were found in some Actinobacteria, but not found in *Mycobacterium tuberculosis*. ‘Bacteroid. PolY2’ polymerases are mostly second Y family polymerases in some Bacteroidetes bacteria (e.g., *Beliella baltica* DinB (AFL82914.1)). A small group that includes Y family polymerases from diverse phyla was called ‘acPolY2-like’ based on second active Y family polymerase of *Acidobacterium capsulatum* (ACO33562.1). The ‘ε-prot PolIV-like’ group encompasses the majority of Y family polymerases found in ε-proteobacteria (e.g., *Arcobacter nitrofigilis* PolIV (ADG92494.1)). At least two subgroups can be discerned from other (than ImuB) inactive PolY’s: ‘scDinB2-like’, a group encompassing only *Streptomyces* inactive PolY’s, represented by *Streptomyces coelicolor* DinB2 (CAB50953.1); ‘gbDinB2-like’ – a group encompassing inactive PolY’s from Spirochaetes, Nitrospirae, δ-proteobacteria, represented by *Geobacter bemidjiensis* DinB2 (ACH37529.1).

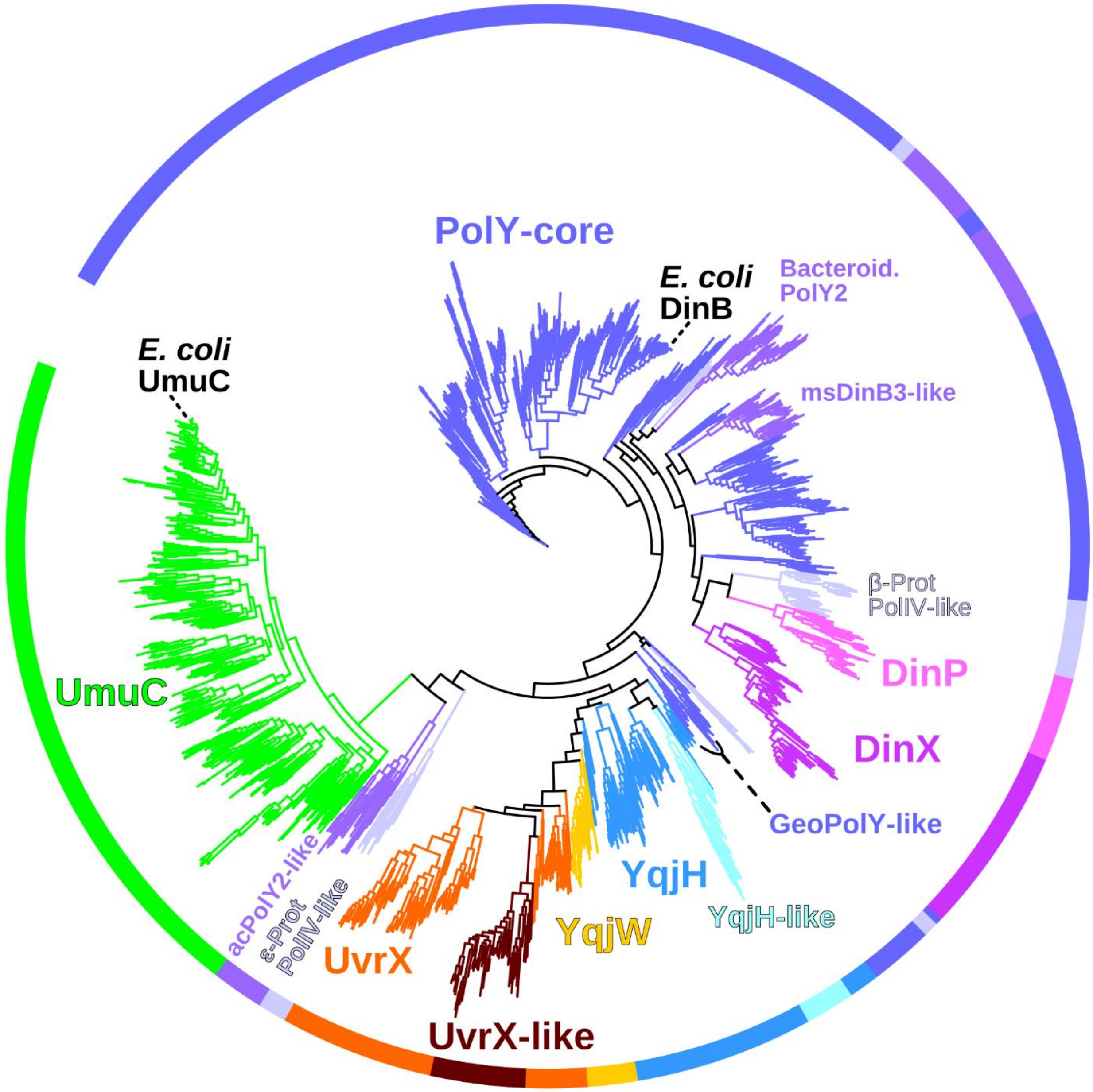

Supplementary Figure S2. Phylogenetic tree constructed for active Y family polymerases. All marked groups can be confidently separated (bootstrap support values >99%). The root of the tree is arbitrary. Positions of *Escherichia coli* DinB and UmuC polymerases are indicated in black. 'β-Prot PolIV-like' is a group of PolY's separated from PolY-core and found in some Betaproteobacteria (e.g., *Paraburkholderia xenvorans* DinB, accession id: ABE33920.1). Also indicated is the position of the small subgroup of PolY-core, 'GeoPolY-like', represented by polymerases from the *Geobacter* family bacteria (e.g., *Geobacter sulfurreducens* DinB1 (id: AAR34990.1) and DinB2 (id: AAR33378.1)), that have conserved C-terminal motifs (see also Supplementary Figure S10).

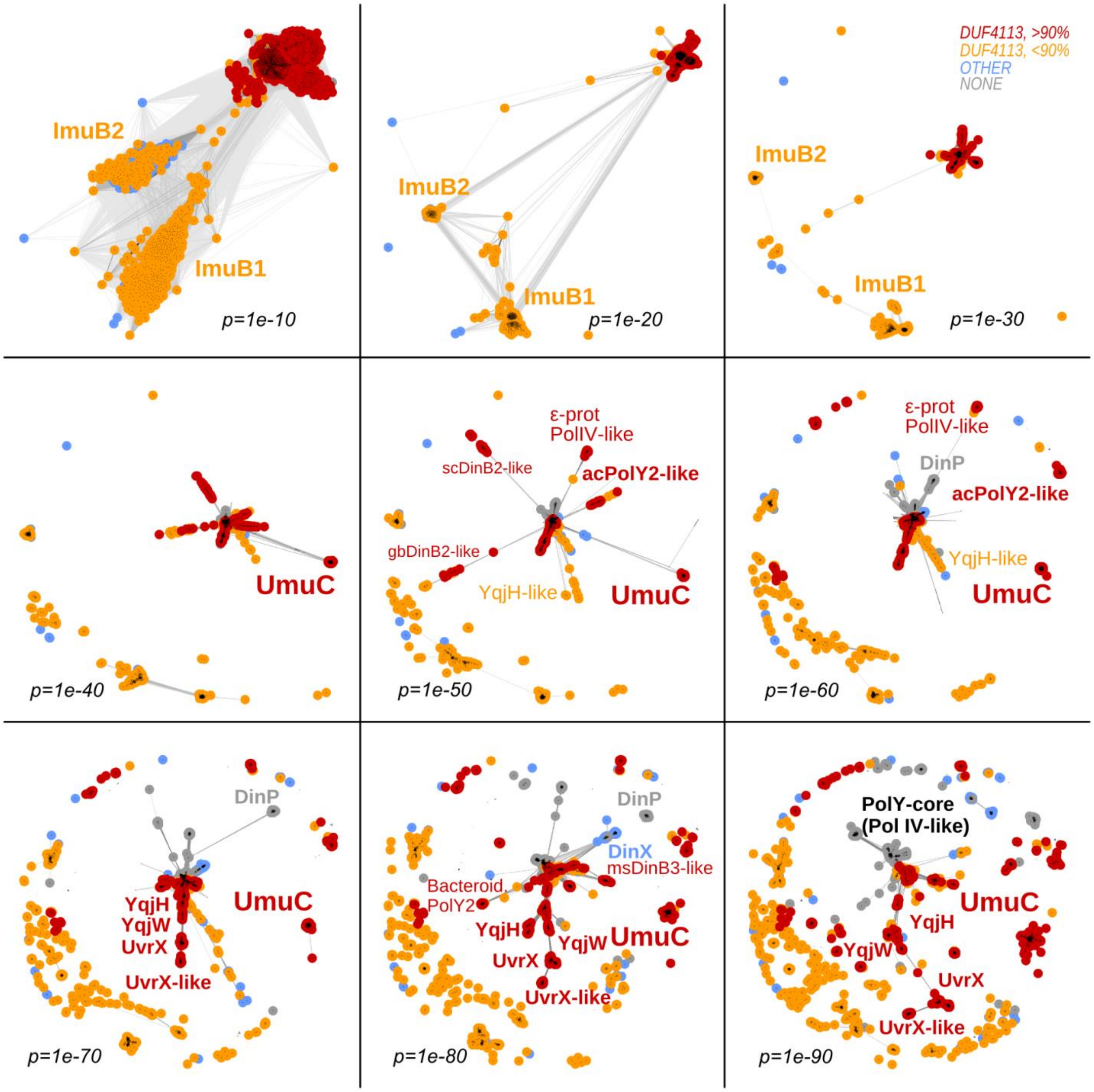

Supplementary Figure S3. Distribution of DUF4113-containing C-terminals among Y family polymerases, depicted in 9 snapshots of Y family clustering series. Clustering snapshots correspond to the Supplementary Figure S1. Sequences are colored based on DUF4113 detection results: red represents highly confident results, orange – less confident results, blue – PolY's with C-terminals, but no identified DUF4113 and grey – PolY's with no additional C-terminal sequences at all.

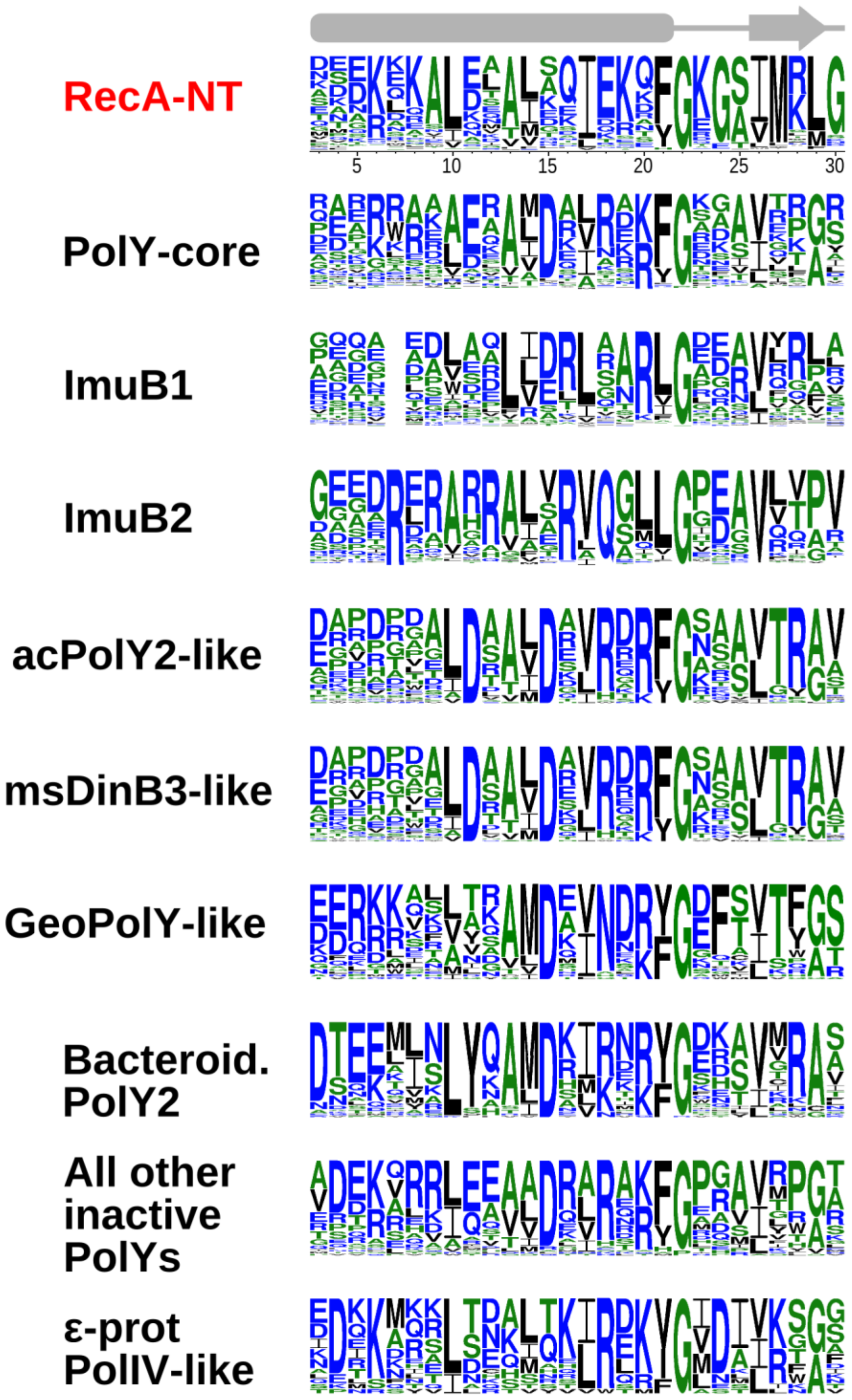

Supplementary Figure S4. Sequence logo comparison of RecA-NT motif and RecA-NT-like motifs of other PolY's (see Figure 4 for motifs of UmuC, YqjW, UvrX(-like) and YqjH).

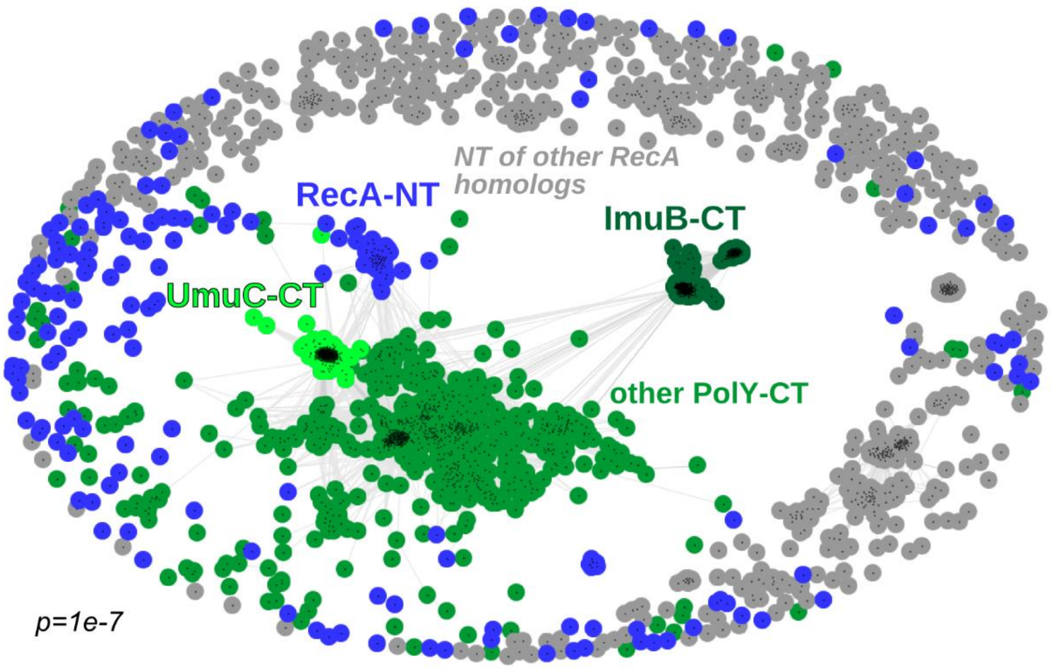

Supplementary Figure S5. Clustering of N-terminal regions of bacterial RecA homologs (blue and grey) together with C-terminal regions of Y family polymerase groups (green), that were identified to have a DUF4113 motif. Identified bacterial RecA are colored blue, other RecA homologs (such as ImuA, SulA, bacterial RadA, KaiC, archaeal RadA, eukaryotic Rad51) are colored grey. RecA homologs were identified by searching with *E. coli* RecA sequence through Uniref50 database using PSI-BLAST. The resulting set had few ImuA homologs, so it was enriched with ImuA (and SulA) sequence groups identified in bacterial proteomes. Full length RecA homolog sequences were clustered together and identifiable known groups separated (unconnected sequences discarded) before cutting and adding their NT's to the set for this figure. All sequences that formed a cluster with *E. coli* RecA at p-value cutoff of 1e-45 were considered to be RecA (blue).

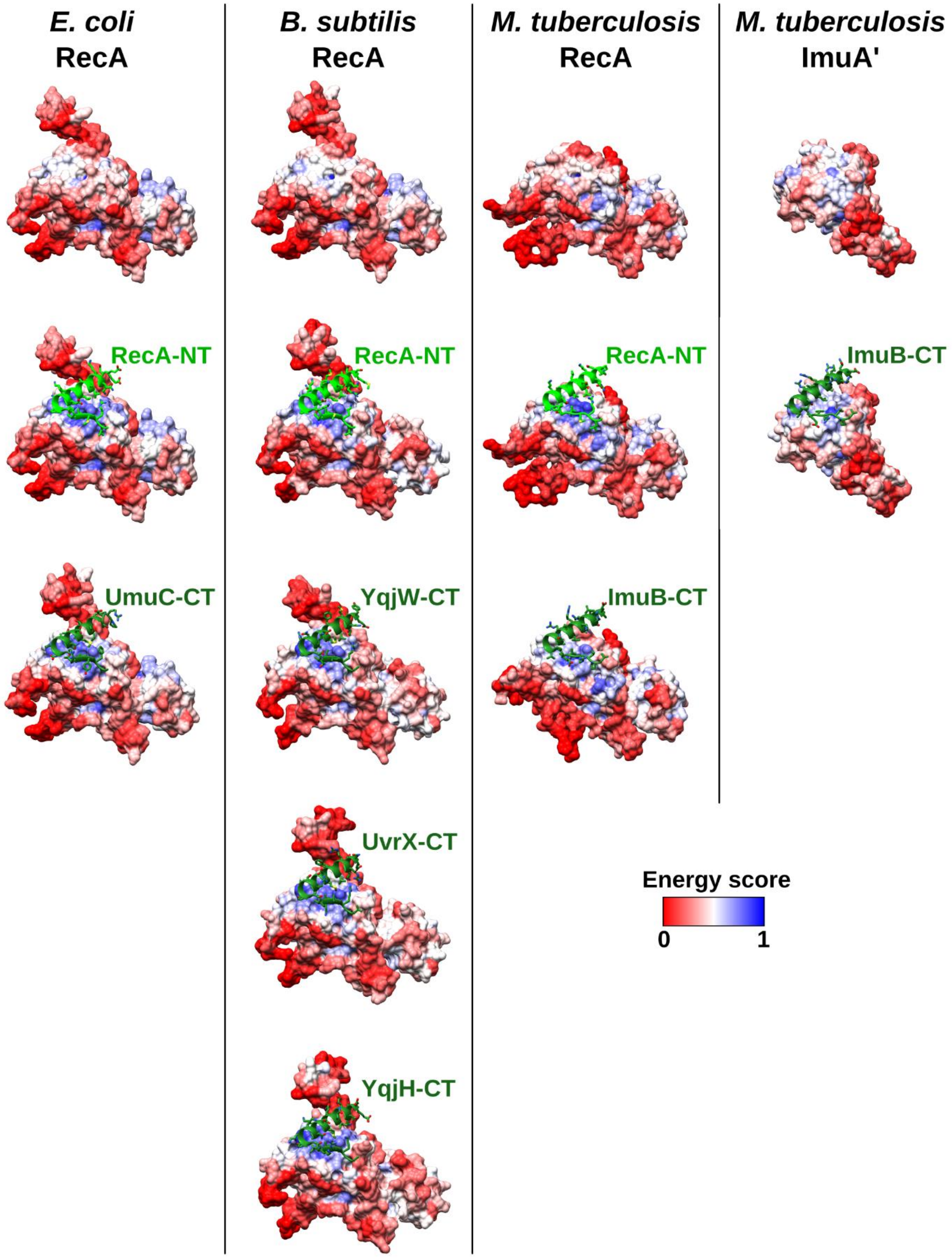

Supplementary Figure S6. Energetically favorable interactions for various motif complexes with RecA or ImuA’ depicted as colored surfaces (blue color represents the most favorable surfaces). Energy scores calculated with VoroMQA.

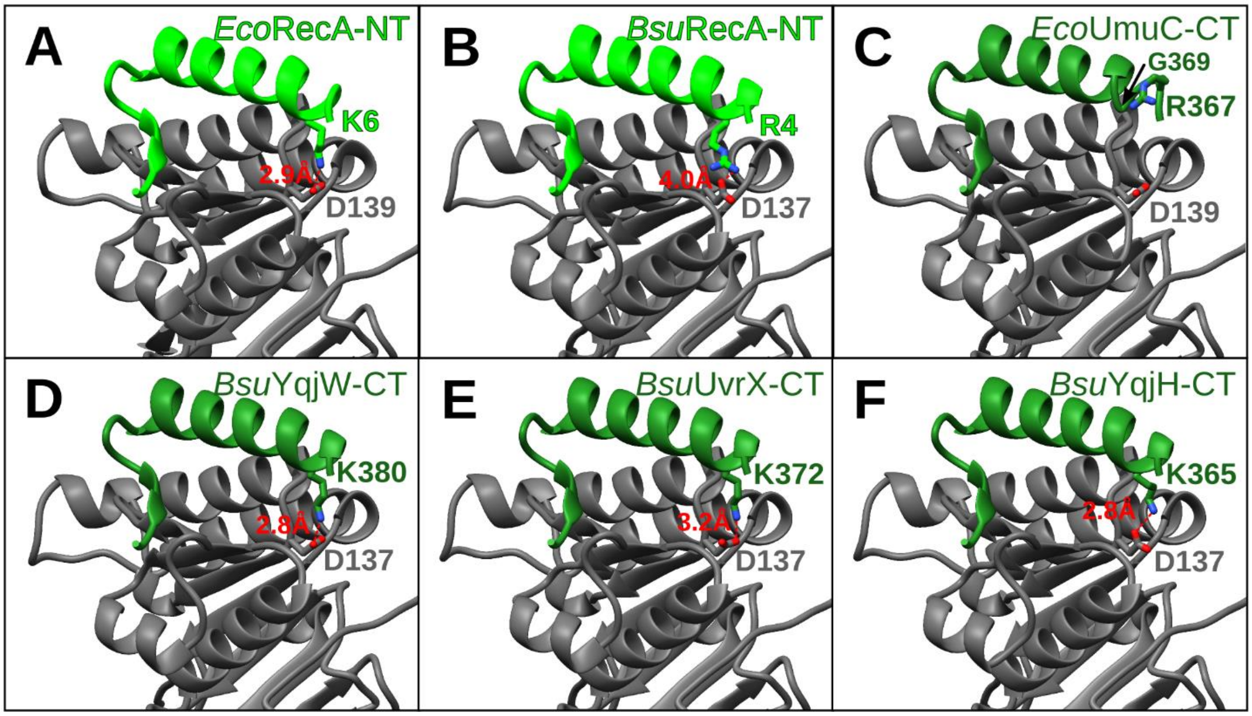

Supplementary Figure S7. A salt bridge, observed in RecA-RecA interface (A – *E. coli*, B – *B. subtilis*) at the first half of α-helix of the RecA-NT motif. The salt bridge in corresponding site in *E. coli* RecA-UmuC model is not observed (C), but is observed in *B. subtilis* PolY-CT models with RecA (D, E, F).

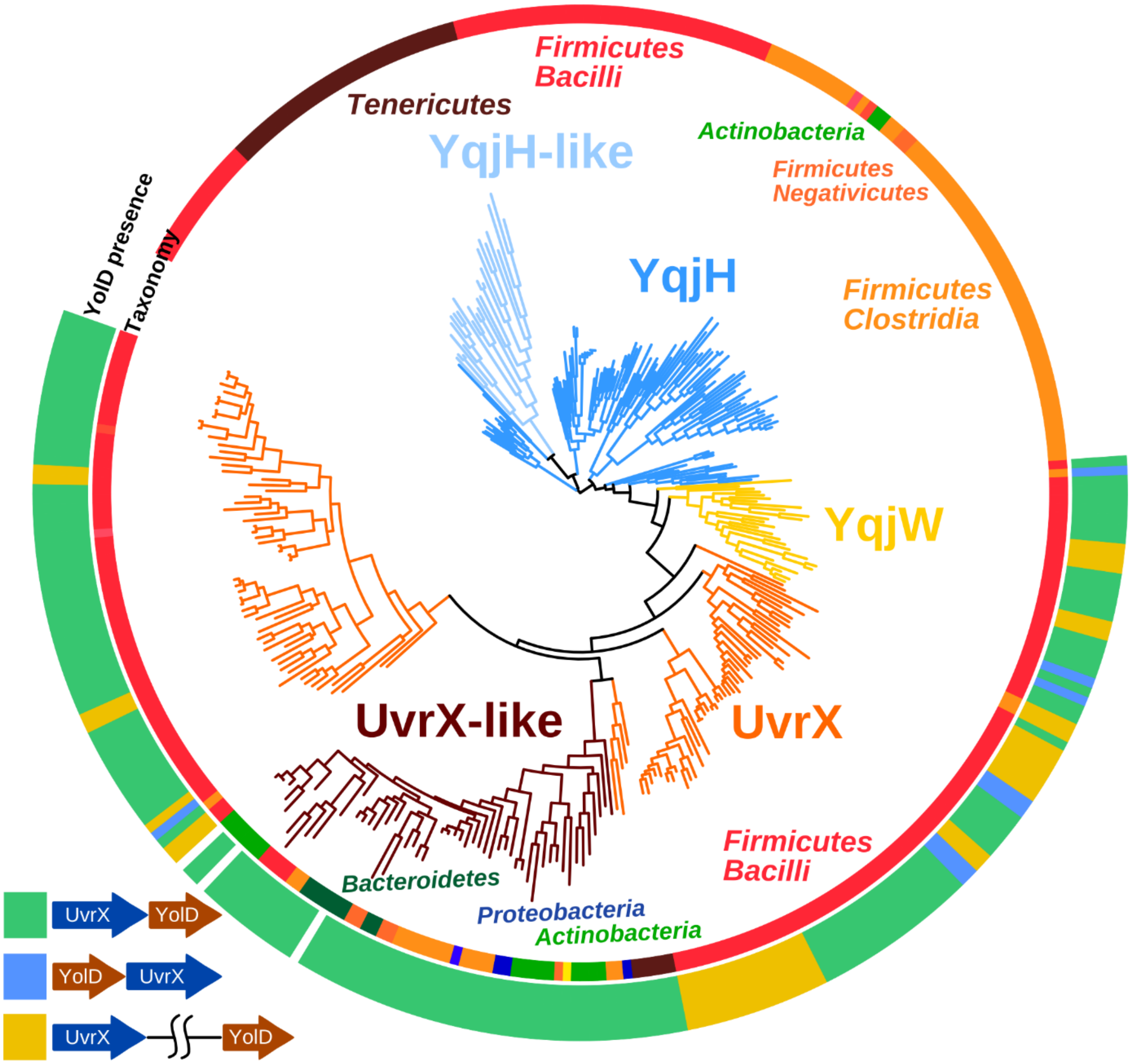

Supplementary Figure S8. Phylogenetic tree of YqjH, YqjW, UvrX and UvrX-like groups. Taxonomic distribution is depicted as inner color strip. Presence of YolD homolog and its relative position to the corresponding PolY is depicted as the outer color strip: green represents PolY-YolD operon (typical), blue – YolD-PolY operon (inverted) and yellow represents cases where both PolY and YolD are present in the genome, but not coded adjacently (split). The presence of YolD homologs in genomes together with YqjH/YqjH-like is not shown, because not a single YqjH form an operon with YolD. The only cases of YolD homolog present together with YqjH/YqjH-like protein in the genome are where UvrX/YqjW is also present in operon with the YolD.

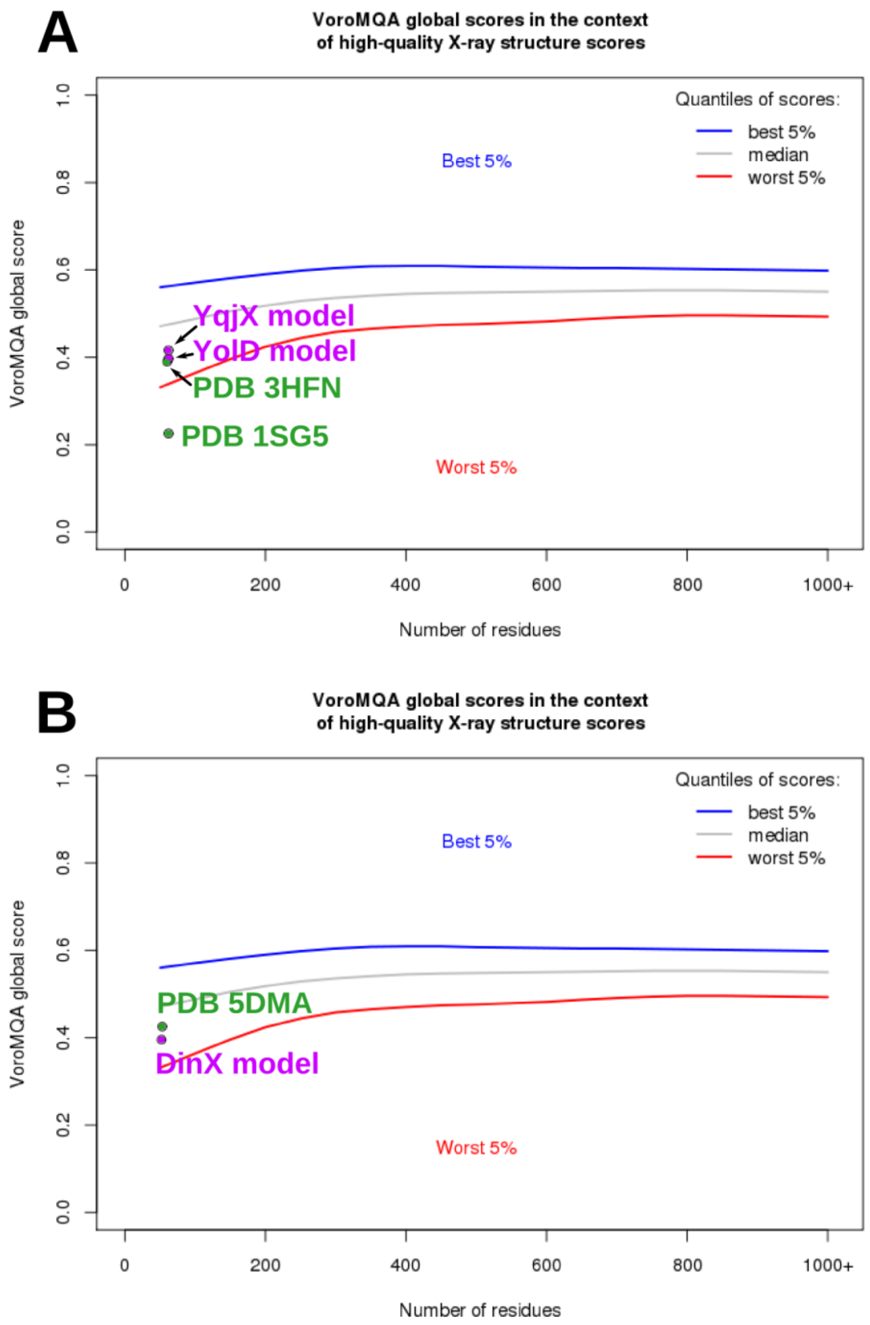

Supplementary Figure S9. Comparison of model and template quality scores (calculated with VoroMQA). The distribution of all high quality X-ray structure scores is also depicted for comparison. A) Comparison of *B. subtilis* YqjX and YolD partial models (accession ids CAB14303.1 and CAB14068.2, respectively) and two best scoring templates, identified with HHpred (*Nostoc sp.* Hfq protein, PDB id 3HFN, and solution structure of *E. coli* Yaeo inhibitor of transcription termination, PDB id 1SG5). B) Comparison of model of *M. tuberculosis* DinX Tudor-like domain (accession id CCP44301.1) and best template used for modeling (Tudor domain of *G. stearothermophilus* PcrA/UvrD helicase, PDB id 5DMA).

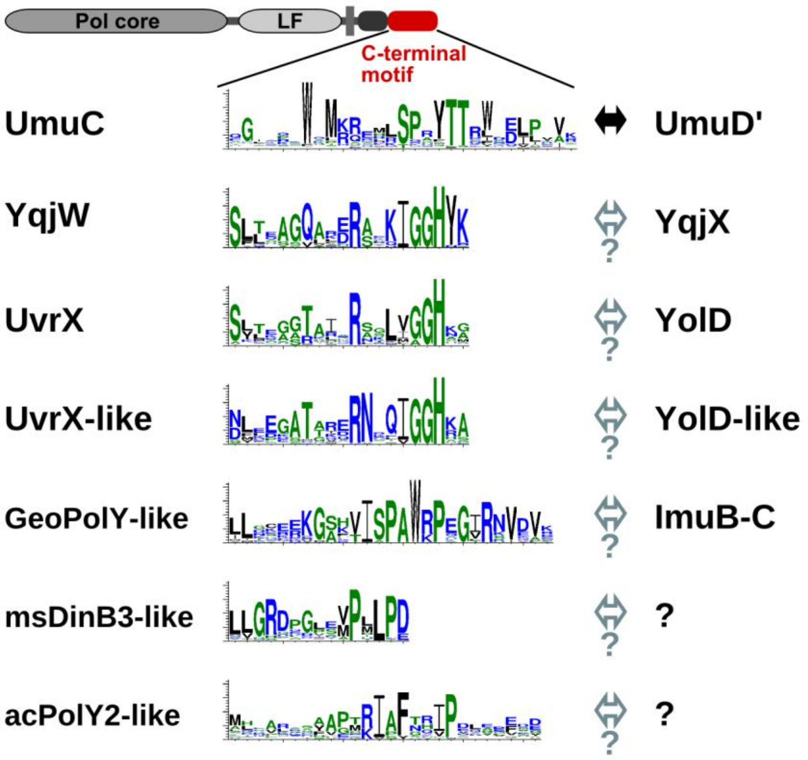

Supplementary Figure S10. Conserved additional C-terminal motifs of bacterial Y family DNA polymerase subfamilies, depicted as sequence logos. Known and predicted interaction partners are listed to the right of each motif. The GeoPolY-like group is a small subset of the PolY-core group, represented by PolY's from *Geobacter* family bacteria. GeoPolY-like C-terminal tails may interact with ImuB-C proteins that are coded together in the genome; however, no additional data to support such putative interaction is available. Other ImuB-C containing PolY groups have different or unconserved C-terminal tails. Two other groups, msDinB3-like and acPolY2-like, have conserved C-terminal tails, but no suggestion for putative interaction partner can be made from our data.
